# Palladium(0) catalyzed Suzuki cross-coupling reaction of 2,5-dibromo-3-methylthiophene: selectivity, characterization, DFT studies and their biological evaluations

**DOI:** 10.1186/s13065-018-0404-7

**Published:** 2018-05-04

**Authors:** Komal Rizwan, Muhammad Zubair, Nasir Rasool, Tariq Mahmood, Khurshid Ayub, Noorjahan Banu Alitheen, Muhammad Nazirul Mubin Aziz, Muhammad Nadeem Akhtar, Faiz-ul-Hassan Nasim, Snober Mona Bukhary, Viqar Uddin Ahmad, Mubeen Rani

**Affiliations:** 1 Department of Chemistry, Government College University, Faisalabad, 38000 Pakistan; 2 Department of Chemistry, Government College Women University, Faisalabad, Pakistan; 3 Department of Chemistry, COMSATS Institute of Information Technology, University Road, Tobe Camp, Abbottabad, 22060 Pakistan; 4 Deparment of Cell and Molecular Biology, Faculty of Biotechnology and Biomolecular Science, University Putra Malaysia, 43400 Serdang, Selangor Darul Ehsan Malaysia; 5 Faculty of Industrial Sciences & Technology, University Malaysia Pahang, Lebuhraya Tun Razak, 26300 Kuantan, Pahang Malaysia; 6 Department of Chemistry, The Islamia University of Bahawalpur, Bahawalpur, 63000 Pakistan; 7 HEJ Research Institute of Chemistry, International Centre for Chemical and Biological Sciences, University of Karachi, Karachi, Pakistan

**Keywords:** Density functional theory, Thiophene, Antioxidant, Antibacterial, Palladium

## Abstract

Thiophene derivatives have shown versatile pharmacological activities. The Suzuki reaction proved a convenient method for C–C bond formations in organic molecules. In the present research work novel derivatives of 2,5-dibromo-3-methylthiophene (**3a**–**k** and **3l**–**p**) has been synthesized, via Suzuki coupling reaction in low to moderate yields. A wide range of functional groups were well tolerated in reaction. Density functional theory investigations on all synthesized derivatives (**3a**–**3p**) were performed in order to explore the structural properties. The pharmaceutical potential of synthesized compounds was investigated through various bioassays (antioxidant, antibacterial, antiurease activities). The compounds **3l**, **3g**, **3j**, showed excellent antioxidant activity (86.0, 82.0, 81.3%), respectively by scavenging DPPH. Synthesized compounds showed promising antibacterial activity against tested strains. **3b**, **3k**, **3a**, **3d** and **3j** showed potential antiurease activity with 67.7, 64.2, 58.8, 54.7 and 52.1% inhibition at 50 µg/ml. Results indicated that synthesized molecules could be a potential source of pharmaceutical agents.

**Electronic supplementary material:**

The online version of this article (10.1186/s13065-018-0404-7) contains supplementary material, which is available to authorized users.

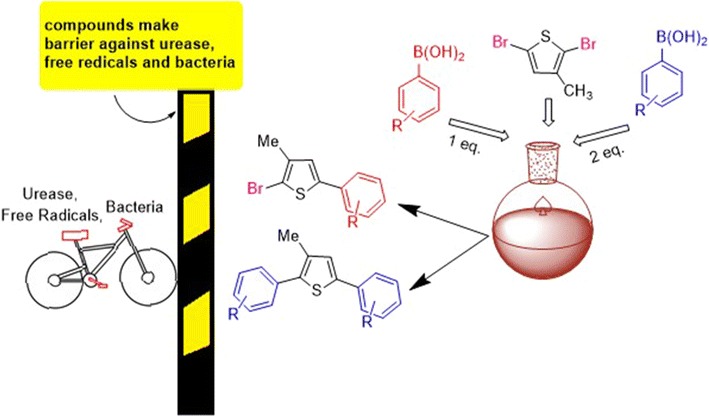

## Background

Thiophene is found in central core of various compounds and is well known for its intrinsic electronic properties [1, 2]. A number of thiophene based heterocycles have been reported for versatile pharmacological activities [3–9]. Biaryl thiophenes are pharmacologically important agents and widely used as anti-inflammatory [10], chemotherapeutic [11], antimicrobial [12] and antioxidant agents [13]. Several reports about regioselective Suzuki coupling of dibromothiophene are available in literature [14, 15]. Palladium catalyzed coupling of 2,5-dibromothiophene has been reported and the yield of obtained product was low (29%) [16]. Synthesis of 2,5-diheteroarylated thiophenes from 2,5-dibromo thiophene derivatives has been reported in good yield [17]. Regioselective Suzuki coupling of 2,5-dibromo-3-hexylthiophene has been reported and preferably coupling occurred at C5 position [18]. The more electron deficient carbon moiety is preferably reactive towards attacking nucleophiles, whereas other reactive carbons do not show any response. Different heterocycles undergo electrophilic substitutions and this regioselectivity can be applied to these substrates [19]. In heterocycles substitution reactions, heteroatom (O, S and N) electron lone pair is being donated to the ring. However, in halogenated thiophenes Suzuki reaction with high oxidative addition, the arylboronate anion preferably attacks the electron deficient carbon bonded with the halogen. And it was observed that transmetallation step is faster due to negatively charged boronate anion then the neutral boronic acids [20]. Extending the scope of Suzuki coupling reaction in regioselective domain a series of 2,5-dibromo-3-methylthiophene derivatives has been synthesized specially with aim to explore their biological importance for the first time.

## Results and discussion

### Chemistry

**Scheme 1:**
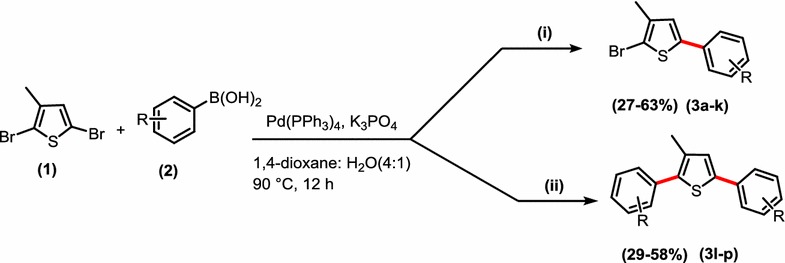
Synthesis of 2-bromo-3-methyl-5-arylthiophenes (**3a**–**k**) and 2,5-diaryl-3-methyl thiophenes (**3l**–**p**). Conditions: (i) 1 (128 mg, 0.5 mmol, 1 eq), **2** (0.55 mmol, 1.1 eq), Pd(PPh_3_)_4_ (14.5 mg, 2.5 mol%), K_3_PO_4_ (212 mg, 1.0 mmol, 2 eq), 1,4-dioxane (2.5 ml), H_2_O (0.625 ml), 12 h, 90 °C under argon. (ii) **1** (128 mg, 0.5 mmol, 1 eq), **2** (1.25 mmol, 2.5 eq,), Pd(PPh_3_)_4_ (34.6 mg, 6 mol%), K_3_PO_4_ (424 mg, 2.0 mmol, 4 eq), 1,4-dioxane (2.5 ml), H_2_O (0.625 ml), 12 h, 90 °C under argon

A series of thiophene derivatives (**3a**–**k**) and (**3l**–**p**) has been synthesized by reaction of 2,5-dibromo-3-methylthiophene with variety of arylboronic acids in low to moderate yields (27–63%) (Scheme 1, Table 1).

**Table 1:**
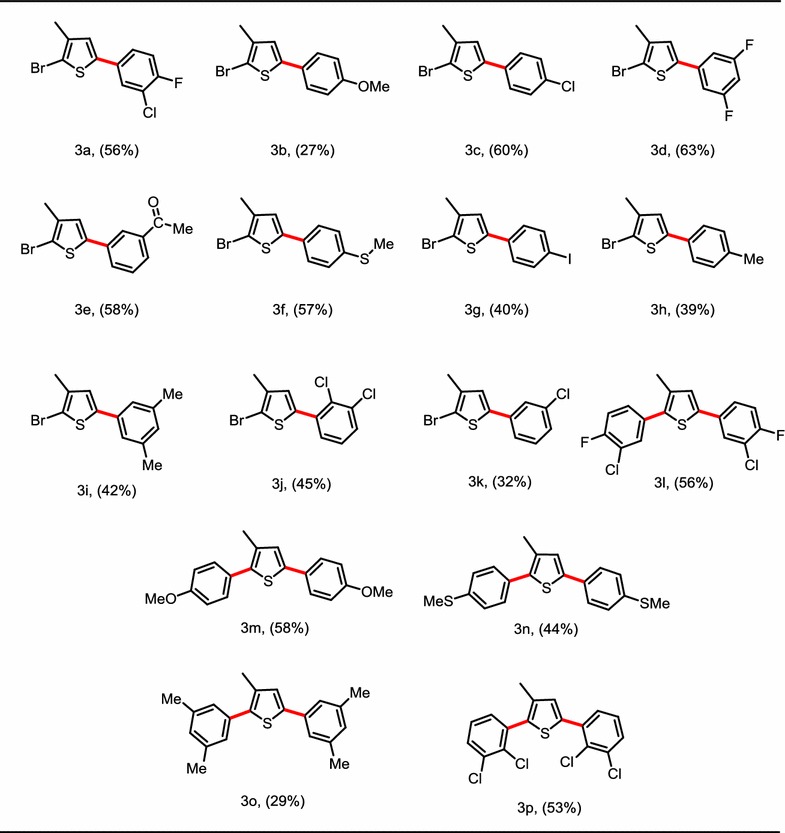
Substrate scope of Suzuki cross coupling reaction of 2,5-dibromo-3-methyl thiophene with variety of arylboronic acids

Under the developed Suzuki reaction conditions, when 1.1 eq of arylboronic acid was used the bromo group at 5 position was selectively substituted and a variety of mono-substituted products was synthesized (**3a**–**k**) and double Suzuki cross coupling occurred by using 2.2 eq of arylboronic acids and diaryl derivatives of thiophene were synthesized (**3l**–**p**) (Table 1). To increase the substrate scope, the arylboronic acids with both electron donating and withdrawing groups were used. The reaction conditions were tolerant of both electron donating and electron withdrawing arylboronic acids. It was noted that some products were obtained in low yield as **3b**, **3h**, **3i**, **3j**, **3k**, **3n**, **3o** which can be attributed to the presence of mixture of mono and di-arylated products in both single and double Suzuki cross coupling reaction and it has been very difficult to separate this reaction mixture and low yields were obtained. This may be due to ineffective transmetallation and reductive elimination in overall reaction cycle [12].

### Density functional theory (DFT) studies

DFT investigations were computed by using GAUSSIAN 09 software, in order to explore the structural properties and reactivity’s of synthesized derivatives. First of all, compounds (**3a**–**3p**) were optimized by using B3LYP/6-31G(d,p) basis set along with the frequency analysis. After optimization the energy minimized structures were used further for frontier molecular orbitals and molecular electrostatic potential (MEP) analysis on the same basis set.

### Frontier molecular orbitals (FMOs) analysis

**Fig. 1:**
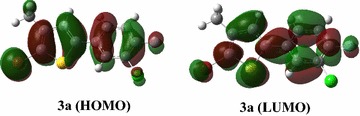
HOMO/LUMO surfaces of compounds (**3a**)

Nowadays frontier molecular orbitals analysis is well known to explain the reactivity of compounds [21] by using different computational methods. The HOMO/LUMO band gap has direct correlation with the reactivity, e.g. if the band is less the compound will be kinetically less stable (more reactive) and vice versa [22]. The FMOs analysis of all derivatives (**3a**–**3p**) was carried out by using B3LYP/6-31G(d,p) basis set. As observed from the HOMO/LUMO, the trend of dispersion of isodensity was almost similar in all compounds. Therefore, as a model here we have given the HOMO/LUMO surfaces of compound **3a** only (Fig. 1) (the rest are provided in Additional file 1: Figure S1). The corresponding HOMO and LUMO energies along with band gap are narrated in Table 2.

**Table 2:** HOMO and LUMO energies, along with band gap

Compounds no	E_HOMO_ (eV)	E_LUMO_ (eV)	ΔE (eV)
**3a**	− 5.93	− 1.39	4.54
**3b**	− 5.39	− 0.92	4.47
**3c**	− 5.83	− 1.38	4.45
**3d**	− 5.99	− 1.50	4.49
**3e**	− 5.84	− 1.69	4.15
**3f**	− 5.39	− 1.12	4.26
**3g**	− 6.08	− 1.77	4.31
**3h**	− 5.60	− 1.06	4.52
**3i**	− 5.60	− 1.05	4.55
**3j**	− 6.04	− 1.43	4.61
**3k**	− 5.91	− 1.40	4.50
**3l**	− 5.81	− 1.59	4.21
**3m**	− 4.98	− 0.86	4.12
**3n**	− 5.06	− 1.16	3.89
**3o**	− 5.24	− 1.04	4.19
**3p**	6.05	− 1.38	4.67

The isodensity in HOMO of all compounds is dispersed on the benzene and thiophene moieties along with the groups attached to the main skeleton. It is clearly reflected from Fig. 1, that in HOMO orbitals the methyl group attached to the thiophene ring and the groups attached to the para position are directly involved in electronic cloud and electronic transition. Whereas isodensity in LUMO of all compounds reflected the similar trend, the methyl attached to thiophene ring and groups attached to the ortho position of benzene did not participate in electronic cloud. The HOMO–LUMO band gap in all compounds found in the range 3.89–4.67 eV. The smallest band gap observed for **3n** i.e. 3.89 eV and largest band gap observed for **3p** i.e. 4.67 eV. HOMO–LUMO band gap is reflecting that **3n** is most reactive and less stable among all, whereas **3p** is most stable and less reactive. This is might be that **3n** has more planer structure, due to which transition of electrons is more feasible, whereas in **3p** the structure is non-planer and does not facilitating the promotion of electrons to higher orbitals easily.

### Molecular electrostatic potential (MEP)

**Fig. 2:**
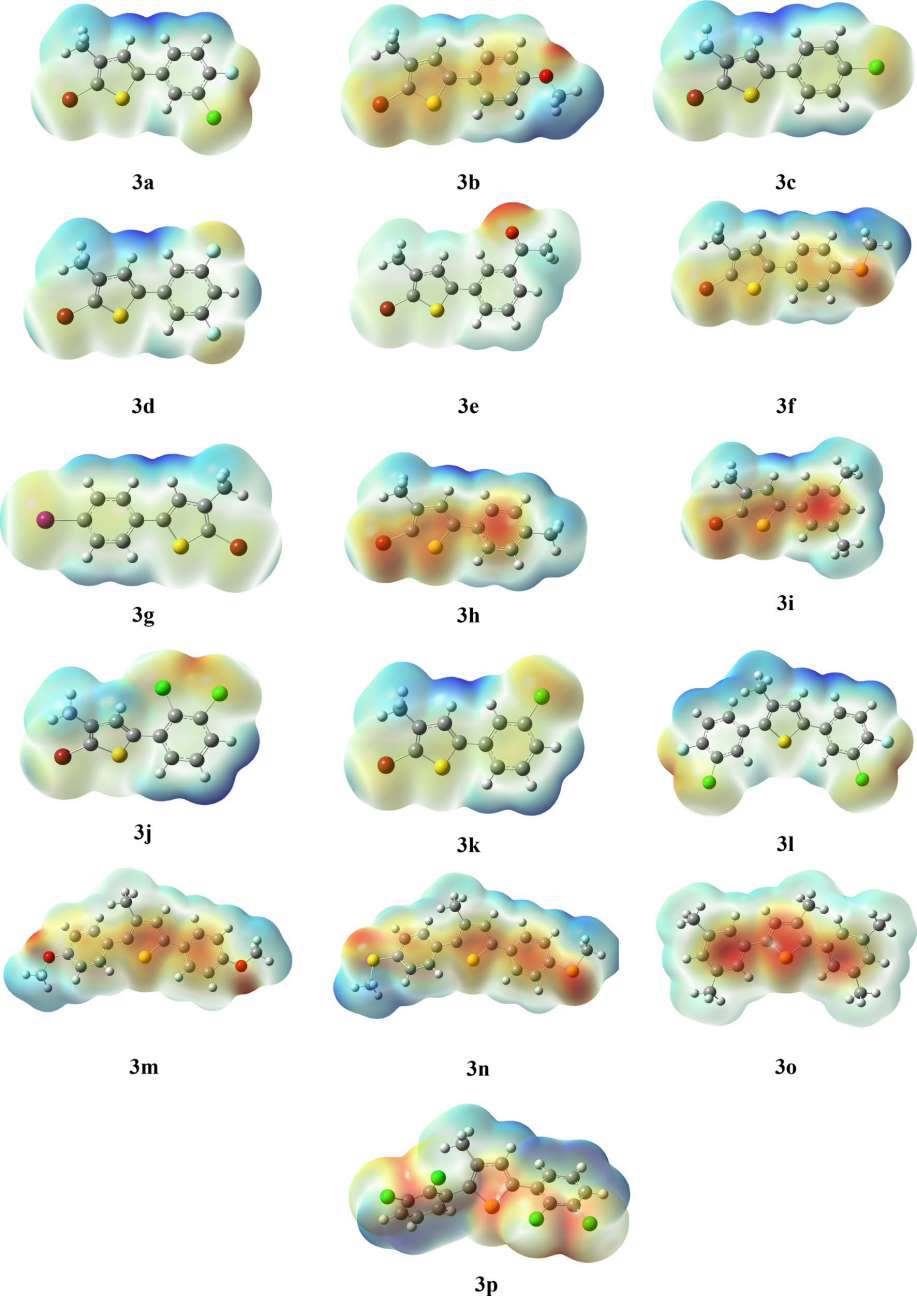
ESP maps of compounds **3a**–**3p**, calculated at DFT/B3LYP/6-31G(d,p) level

Molecular electrostatic potential study by using quantum chemical tools is useful to explain reactivity, charge separations and monovalent interactions of molecules [23]. ESP analysis of compounds **3a**–**3p** was computed by using DFT/B3LYP/6-31G(d,p) basis and graphics (Fig. 2). The range of MEP values of all compounds are given in Additional file 1: Table S1.

In ESP analysis, the dispersion of electronic density is explained on the basis of different colors e.g. the red color indicates the –ve potential and blue color is indicative of +ve potential [24]. It is cleared from ESP analysis that the electronic density in every compound is dispersed with respect to the electronic effect of group attached to the benzene moiety. The groups attached to the para position of benzene ring have direct effect on the electronic cloud of whole molecule. In **3a**, the electron withdrawing group (fluoro) is attached to the benzene ring, due to which the –ve potential is dispersed bromo, chloro and fluoro groups instead of concentrating on benzene ring. Whereas in **3b** the –ve potential is concentrated on benzene and thiophene ring due to electron donating effect of –OCH_3_ attached to the para position on benzene ring. Almost similar kind of effect is observed in ESP analysis of all other synthesized derivatives. If electron donating group is attached to the ortho or para position of benzene moiety the electronic density is concentrated on the benzene and thiophene rings (rather the electronic density also depends on the electron donating ability of group as well), such as in compounds **3c**, **3f**, **3g**, **3h**, **3i**, **3k**, **3m**, **3n**, **3o** and **3p**. In all these molecules the –ve potential is concentrated on the benzene and thiophen rings, whereas in the rest of molecules the –ve potential is concentrated on the different groups attached at the different positions of scaffolds (Fig. 2).

### Antioxidant activity by DPPH radical scavenging assay

Antioxidants have been broadly studied for their capability to protect cells and organisms from the harm induced by reactive oxidative species (ROS) [25, 26]. So, scientists are more interested to find sources for antioxidants which may be either natural or synthetic.

The DPPH radical has been widely used for determining antioxidant activity of various systems [27]. DPPH radical is purple in colour and antioxidants decay that purple colour of DPPH by capturing free radicals. The potential of DPPH scavenging can be quantified by noting absorbance at 517 nm. A study was designed to determine the antioxidant potential of some novel thiophene derivatives (**3a**–**k** and **3l**–**p**), by DPPH radical scavenging assay (Table 3). Ascorbic acid was used as control which exhibited 100% DPPH scavenging at 50 µg/ml. The compounds **3l**, **3g**, **3j**, showed excellent antioxidant activity (86.0, 82.0, and 81.3%), respectively by scavenging DPPH. It is noted that some compounds (**3d**, **3n**) showed mild antioxidant activity with 48.2, 40.9% DPPH radical scavenging at 50 µg/ml. However other compounds showed significant antioxidant activity by scavenging DPPH while some compounds exhibited low activity (Table 3). Mabkhot and coworkers found some thiophene moiety containing compounds inactive towards scavenging DPPH and proved them poor antioxidants [28]. The substituents on ring system have pronounced effect on DPPH radical scavenging [29]. So, in light of this reference, this may be cause of variability in DPPH radical scavenging of thiophene based compounds.

**Table 3:** Antioxidant potential of compounds (**3a**–**k** and **3l**–**p**) by DPPH radical scavenging activity

Entry	Compounds no	Percentage inhibition at 50 µg/ml
1	**3a**	33.4 ± 0.29
2	**3b**	23.9 ± 0.31
3	**3c**	37.5 ± 0.42
4	**3d**	48.2 ± 0.42
5	**3e**	38.5 ± 0.42
6	**3f**	39.2 ± 0.42
7	**3g**	82.0 ± 0.78
8	**3h**	***
9	**3i**	28.9 ± 0.45
10	**3j**	81.3 ± 0.72
11	**3k**	21.9 ± 0.32
12	**3l**	86.0 ± 0.73
13	**3m**	1.19 ± 0.02
14	**3n**	40.9 ± 0.21
15	**3o**	15.1 ± 0.21
16	**3p**	30.9 ± 0.29
17	Ascorbic acid	100 ± 0.99

*** Showed no activity. The results are average ± SD of triplicate experiments *p* < 0.05

### Antibacterial activity

Thiophene and its various derivatives have been reported for potential anti-microbial activity [30–32]. To overcome the drug resistance issues it is very important to develop new anti-microbial agents. Generally in the field of pharmaceutical, new drugs are developed by molecular modification of well-known compounds whose activity is already established. So a novel series of thiophene derivatives (**3a**–**k** and **3l**–**p**) were screened for anti-bacterial activity against variety of Gram-positive and Gram-negative bacterial strains. Percentage inhibition of bacterial growth was examined at concentration (50 μg/ml). For examining the antibacterial activity of series **3a**–**k** and **3l**–**p**, streptomycin was used as standard drug which showed 100% inhibition against various bacterial strains (Table 4). Compounds **3a**, **3k**, **3i** showed highest activity against *P. aeruginosa* with % inhibition 67.3, 50.5, 41.1% at 50 μg/ml while compounds **3b**, **3h**, **3d** and **3n** showed moderate activity with 39.2, 37.6, 34.9, 20.8% inhibition. This series of thiophene compounds did not show any activity against *B. subtilis*. When activity was observed against *E. coli* compounds **3a**, **3k**, **3i** showed excellent activity with 94.5, 72.5, 70.4% inhibition. While **3b**, **3h** and **3n** showed moderate inhibitory effect against *E. coli*. Compound **3a** and **3k** showed moderate activity against *S. aureus* and *S. typhimurium* while compound **3b** and **3i** showed low activity against these two strains. It was observed that compounds **3c**, **3e**, **3f**, **3g**, **3j**, **3l**, **3m**, **3o** and **3p** were found inactive against *P. aeruginosa, B. subtilis, E. coli, S. aureus* and *S. typhi* (Table 4).

**Table 4:** Antibacterial activity of synthesized compounds (**3a**–**k** and **3l**–**p**) against Gram positive and Gram negative bacteria

Entry	Product no	% inhibition (50 μg/ml)				
		*P. aeruginosa*	*B. subtilis*	*E. coli*	*S. aureus*	*S. typhi*
1	**3a**	67.3 ± 0.76	***	94.5 ± 0.09	33.9 ± 0.37	27.6 ± 0.08
2	**3b**	39.2 ± 0.45	***	50.1 ± 0.29	9.57 ± 0.15	5.58 ± 0.05
3	**3c**	***	***	***	***	***
4	**3d**	34.9 ± 0.27	***	7.8 ± 0.09	***	8.34 ± 0.23
5	**3e**	***	***	***	***	**
6	**3f**	***	***	***	***	***
7	**3g**	***	***	***	***	***
8	**3h**	37.6 ± 0.26	***	50.4 ± 0.45	***	12.0 ± 0.02
9	**3i**	41.1 ± 0.47	***	70.4 ± 0.78	***	2.59 ± 0.01
10	**3j**	***	***	***	***	***
11	**3k**	50.5 ± 0.58	***	72.5 ± 0.87	20.1 ± 0.06	17.3 ± 0.05
12	**3** **l**	***	***	***	***	***
13	**3m**	***	***	***	***	***
14	**3n**	20.8 ± 0.17	***	30.6 ± 0.26	***	*
15	**3o**	***	***	***	***	***
16	**3p**	***	***	***	***	***
17	Control	100 ± 1.28	100 ± 1.21	100 ± 1.01	100 ± 0.99	100 ± 0.99

*** Showed no activity. The results are average ± SD of triplicate experiments *p* < 0.05. Streptomycin was used as control standard drug

The compounds with both electron donating and withdrawing groups showed good to moderate antibacterial activity. This activity was found promising for future benefits of these compounds as anti-bacterial agents. All the thiophene derivatives that were tested for antibacterial activity were found inactive against *B. subtilis*. Previous reports about substituents effects on anti-microbial activity of thiophene based compounds are available in literature [31–33]. This context is a great deal for researchers to determine the medicinal values of thiophene based compounds.

### Antiurease activity

The metalloenzyme urease involved in catalyzing the hydrolysis of urea. It is present in some plant varieties, algae, microbes and as well in soil enzymes [34]. This enzyme is involved in pathogenesis of various diseases and cause significant environmental and agriculture issues [35]. Several compounds have been reported as urease inhibitors to reduce agriculture, environmental, medical issues and to enhance the uptake of urea [36]. Heteroaryl pharmacophores have potential inhibitory activity against bacterial and plant urease [37]. A library novel of thiophene based compounds (**3a**–**k**, **3l**–**p**) were screened for antiurease activity (Table 5), where thiourea was used as positive control and it showed 98.3% urease inhibition at 50 µg/ml. From these series of thiophene compounds **3b**, **3k**, **3a**, **3d** and **3j** showed potential antiurease activity with 67.7, 64.2, 58.8, 54.7 and 52.1% inhibition at 50 µg/ml. It was noted that some compounds **3c**, **3e**, **3f**, **3g**, **3h** and **3i** showed moderate antiurease activity. Some of the novel synthesized products exhibited relatively higher antiurease activity while other products showed moderate urease inhibition effects. It is concluded that compounds with electron donating substituents on aryl ring have pronounced effect on urease inhibition and those compounds showed higher antiurease activity. While compounds with electron withdrawing substituents showed less activity. This may be due to decrease in metal chelating activity caused by electron withdrawing substituents and vice versa. These results are in agreement with previously reported antiurease activity of thiophene based compounds [33–38]. According to previous study chelation/removal of nickle ions resulted in inactivation of the enzyme [39]. Therefore change in electronic environment and position and orientation of functional groups can be attributed to variability in antiurease activity of different compounds.

**Table 5:** Antiurease activity of synthesized compounds (**3a**–**k** and **3l**–**p)**

Entry	Compound no	Percentage inhibition at 50 µg/ml
1	**3a**	58.8 ± 0.58
2	**3b**	67.7 ± 0.77
3	**3c**	48.9 ± 0.65
4	**3d**	54.7 ± 0.67
5	**3e**	42.9 ± 0.45
6	**3f**	40.3 ± 0.40
7	**3g**	34.2 ± 0.38
8	**3h**	38.8 ± 0.45
9	**3i**	36.9 ± 0.45
10	**3j**	52.1 ± 0.78
11	**3k**	64.2 ± 0.87
12	Thiourea	95.6 ± 0.87

The results are average ± SD of triplicate experiments *p* < 0.05. Thiourea used as positive control

## Methods

### General

The starting materials were purchased from Fisher Scientific company (Pittsburgh, PA, USA) and Sigma Aldrich Chemical Company (St. Louis MO, USA). Characterization of compounds was done by ^1^H, ^13^C NMR Spectra, and melting point determination (for solids). ^1^H, ^13^C, NMR Spectra at 500, 126, MHz, respectively. Melting points (°C) were recorded of solid compounds. TLC silica gel plates (0.25 mm) were used for monitoring the reaction. Ultraviolet light (UV) was used for visualization. Spectrometer JMS-HX-110 equipped with a data system was used for recording the EI/MS spectra. For elemental analysis CHNS/O analyzer (Perkin-Elmer 2400 series) was used. Silica gel of various mesh sizes was used (70–230 mesh and 30–400 mesh).

### General procedure for synthesis of **3a**–**k** and **3l**–**p**

In a reaction vial stirring bar, catalyst Pd(PPh_3_)_4_, 2,5-dibromo-3-methylthiophene (1 eq) was added. A disposable Teflon septum was used to seal vial, which was first evacuated, then purged with argon thrice. 1,4-dioxane solvent was added with syringe with stirring under argon. Stirring of mixture was done at rt for 30 min. After that aryl boronic acid, K_3_PO_4_ and water was added [15] and again vial was sealed and purged with argon three times and it was stirred for 12 h at 90 °C, and then cooled to rt. After that, ethyl acetate was used for dilution of mixture, the organic layer was separated and MgSO_4_ was used for drying this layer and through the vacuum the remaining solvent was evaporated. The purification of crude product was done by the column chromatography by using ethyl-acetate and *n*-hexane (0–50% gradient) to obtain the desired compounds.

### Characterization data

#### 2-Bromo-5-(3-chloro-4-fluorophenyl)-3-methylthiophene (**3a**)

Obtained as a white solid, mp = 113–114 °C, (86 mg, 56%). ^1^H NMR (CD_3_OD, 500 MHz): δ 7.72 (dd, *J *= 6.5, 2.4 Hz, 1H-aryl), 7.56–7.54 (m, 1H-aryl), 7.33–7.30 (m, 1H-aryl, 1H-thiophene), 1.28 (s, 3H-Me); ^13^C NMR (CD_3_OD, 126 MHz): δ 110.0, 109.8, 117.0, 121.3, 127.3 (2C), 129.2, 130.5, 141.2, 142.3, 158.9, EI/MS *m/z* (%): 304.9 [M+H]; 305.5 [M+2, 130.0]; 307.5 [M+4, 31.0]; [M-Me] = 289.0; [M-Me, Br] = 210.5. Anal. Calcd. For C_11_H_7_BrClSF: C, 42.14; H, 2.42; Found: C, 42.50; H, 2.68%.

#### 2-Bromo-5-(4-methoxyphenyl)-3-methylthiophene (**3b**)

Obtained as a brown solid, mp = 98–99 °C, (38 mg, 27%). ^1^H NMR (CD_3_OD, 500 MHz): δ 7.45 (d, *J *= 9.0 Hz, 2H-Aryl), 6.88 (s, 1H-thiophene), 6.92 (d, *J *= 9.0 Hz, 2H-Aryl), 3.80 (s, 3H-OMe), 2.17 (s, 3H-Me); ^13^C NMR (CD_3_OD, 126 MHz): δ 12.5, 56.8, 110.8, 115.8 (2C), 126.7, 127.8, 128.5 (2C), 141.5, 143.0, 161.6, EI/MS *m/z* (%): 284.1 [M+H]; 285.2 [M+2, 90.5]; [M-Me] = 267.2, [M-Br] = 204.2, [M-Br, Me, OMe]^+^ = 159.0. Anal. Calcd. For C_12_H_11_BrOS: C, 49.9, H, 3.92; Found: C, 50.8, H, 3.98%.

#### 2-Bromo-5-(4-chlorophenyl)-3-methylthiophene (**3c**)

Obtained as a yellow solid, mp = 76–79 °C, (85 mg, 60%). ^1^H NMR (CD_3_OD, 500 MHz): δ 7.58 (d, *J *= 8.7 Hz, 2H-aryl), 7.52 (d, *J *= 8.7 Hz, 2H-aryl), 7.13 (s, 1H- thiophene), 2.18 (s, 3H-Me); ^13^C NMR (CD_3_OD, 126 MHz): δ 12.0, 108.4, 127.5, 128.6 (2C), 129.4 (2C), 131.6, 134.2, 140.2, 142.2. EI/MS *m/z* (%): 288.2 [M+H]; 289.3 [M+2, 130.0]; 291.0 [M+4, 31.8]; [M-Br] = 207.0; [M-Br, Cl fragments] = 172.1. Anal. Calcd. For C_11_H_8_BrClS: C, 45.9; H, 2.80; Found: C, 45.0; H, 2.90%.

#### 2-Bromo-5-(3,5-difluorophenyl)-3-methylthiophene (**3d**)

Obtained as a yellow solid, mp = 78–80 °C, (92 mg, 63%). ^1^H NMR (CD_3_OD, 500 MHz): δ 7.21–6.98 (m, 3H-aryl), 6.25 (s, 1H-thiophene), 2.43 (s, 3H-Me); ^13^C NMR (CD_3_OD, 126 MHz): δ 11.2, 103.5, 109.9 (m), 110.2, 111.2 (2C), 127.9, 136.2, 141.2, 142.3, 165.1 (m). EI/MS *m/z* (%): 290.0 [M+H]; 291 [M+2, 90.5]; [M-2F] = 250.1, [M-Br] = 209.1, [M-2F, aryl fragments] = 175.0. Anal. Calcd. For C_11_H_7_BrF_2_S: C, 44.28; H, 2.38; Found: C, 44.00; H, 2.42%.

#### 1-(3-(5-Bromo-4-methylthiophene-2-yl)phenyl)ethan-1-one (**3e**)

Obtained as a brown semisolid, (85 mg, 58%). ^1^H NMR (CD_3_OD, 500 MHz): δ 8.08 (d, *J *= 1.5 Hz, 1H-aryl), 7.98–7.86 (m, 1H aryl), 7.64–7.55 (m, 2H), 7.38 (s, 1H-thiophene), 2.65 (s, 3H-OMe), 2.35 (s, 3H-Me); ^13^C NMR (CD_3_OD, 126 MHz): δ 12.0, 27.0, 110.6, 126.2, 127.0, 128.6, 129.0, 130.6, 133.7, 137.3, 141.0, 142.5, 197.6. EI/MS *m/z* (%): 296.0 [M+H]; 297.5 [M+2, 95.3]; [M-MeCO] = 250.9, [M-Br] = 216.1. Anal. Calcd. For C_13_H_11_BrOS: C, 51.79; H, 3.76; Found: C, 51.68; H, 4.00%.

#### 2-Bromo-3-methyl-(4-(methylthio)phenyl)thiophene (**3f**)

Obtained as a white solid, mp = 180–181 °C, (85 mg, 57%). ^1^H NMR (CD_3_OD, 500 MHz): δ 7.46 (d, *J *= 8.5 Hz, 2H-Aryl), 7.25 (d, *J *= 10.5 Hz, 2H-Aryl), 7.09 (s, 1H-thiophene), 2.48 (s, 3H-SMe), 2.18 (s, 3H-Me); ^13^C NMR (CD_3_OD, 126 MHz): δ 11.6, 14.8, 110.0, 127.0, 127.3 (2C), 127.7 (2C), 130.1, 139.5, 141.5, 142.0. EI/MS *m/z* (%): 300.9 [M+H]; 301.9 [M+2, 97.5]; [M-Me] = 283.9, [M-SMe] = 252.6, [M-Br] = 219.0. Anal. Calcd. For C_12_H_11_BrS_2_: C, 47.28; H, 3.82; Found: C, 47.50; H, 3.68%.

#### 2-Bromo-5-(4-iodophenyl)-3-methylthiophene (**3g**)

Obtained as off white solid, mp = 149–150 °C, (75 mg, 40%). ^1^H NMR (CD_3_OD, 500 MHz): δ 7.79 (d, *J *= 8.7 Hz, 2H-aryl), 7.71 (d, *J *= 8.7 Hz, 2H-aryl), 6.95 (s, 1H-thiophene), 2.19 (s, 3H-Me); ^13^C NMR (CD_3_OD, 126 MHz): δ 11.5, 94.0, 110.6, 126.5, 129.0 (2C), 132.5, 138.1, 138.2, 141.2, 144.0. EI/MS *m/z* (%): 380.0 [M+H]; 381.0 [M+2, 90.7], [M-Br] = 299.0; [M-Br, Me fragments] = 283.7; [M-I, Br fragments] = 172.2. Anal. Calcd. For C_11_H_8_BrIS: C, 33.68; H, 2.68; Found: C, 33.57; H, 2.23%.

#### 2-Bromo-3-methyl-5-*p*-tolylthiophene (**3h**)

Obtained as a white solid, mp = 110–111 °C, (52 mg, 39%). ^1^H NMR (CD_3_OD, 500 MHz): δ 7.89 (d, *J* = 6.9 Hz, 2H-aryl), 7.58 (d, *J* = 7.2 Hz, 2H-aryl), 6.98 (s, 1H-thiophene), 2.53 (s, 3H-Me), 2.15 (s, 3H-Me); ^13^C NMR (CD_3_OD, 126 MHz): δ 11.4, 21.2, 109.9, 125.2 (2C), 127.0, 129.5 (2C), 130.2, 131.9, 141.2, 142.0. EI/MS *m/z* (%): 268.0 [M+H]; 269.3 [M+2, 96.4]; [M-Br] = 187.0, [M-Me, Br] = 172.0; [M-Br, Me, thiophene] = 91.2. Anal. Calcd. For C_12_H_11_BrS: C, 53.89; H, 3.15; Found: C, 54.4; H, 3.28%.

#### 2-Bromo-5-(3,5-dimethylphenyl)-3-methylthiophene (**3i**)

Obtained as a yellow solid, mp = 120–122 °C, (58 mg, 42%). ^1^H NMR (CD_3_OD, 600 MHz): δ 7.21–7.15 (m, 2H-aryl), 7.07 (s, 1H-thiophene), 6.97–6.91 (m, 1H-aryl), 2.33 (s, 6H–2Me), 2.31 (s, 3H–Me); ^13^C NMR (CD_3_OD, 150 MHz): δ 11.6, 21.0 (2C), 109.5, 127.4 (3C), 130.6, 133.0, 138.5 (2C), 141.0, 142.6. EI/MS *m/z* (%): 282.0 [M+H]; 283.0 [M+2, 93.5]; [M-Me] = 264.5; [M-Me, Br fragments] = 186.1. Anal. Calcd. For C_13_H_13_BrS: C, 54.5; H, 3.66; Found: C, 54.8; H, 4.23%.

#### 2-Bromo-5-(2,3-dichlorophenyl)-3-methylthiophene (**3j**)

Obtained as a brown solid, mp = 103–104 °C, (72 mg, 45%). ^1^H NMR (CD_3_OD, 600 MHz): δ 7.61–7.58 (m, 1H-aryl), 7.52 (dd, *J *= 8.0, 1.5 Hz, 1H-aryl), 7.46 (dd, *J *= 8.0, 1.5 Hz, 1H-aryl), 7.11 (s, 1H-thiophene), 2.20 (s, 3H-Me); ^13^C NMR (CD_3_OD, 150 MHz): δ 11.8, 110.4, 127.1 (2C), 127.8, 130.3, 131.0, 133.6, 137.3, 141.3, 142.0. EI/MS *m/z* (%): 323.0 [M+H]; 324.3 [M+2, 164.3]; 326.0 [M+4, 74.0]; 328.0 [M+6, 10.0]; [M-Br] = 241.0 [M-2Cl, Br fragments] = 171.0. Anal. Calcd. For C_11_H_7_BrCl_2_S: C, 41.0, H, 2.19; Found: C, 41.8, H, 2.42%.

#### 2-Bromo-5-(3-chlorophenyl)-3-methylthiophene (**3k**)

Obtained as a yellow semisolid, (46 mg, 32%). ^1^H NMR (CD_3_OD, 600 MHz): δ 7.63–7.61 (m, 1H-aryl), 7.55–7.52 (m, 2H-aryl), 7.34 (t, *J *= 7.8 Hz, 1H-aryl), 6.96 (s, 1H-thiophene), 2.19 (s, 3H-Me); ^13^C NMR (CD_3_OD, 150 MHz): δ 12.4, 110.4, 124.3, 127.0, 127.8, 128.9, 130.0, 134.0, 135.2, 141.3, 142.0. EI/MS *m/z* (%): 288.0 [M+H]; 289.3 [M+2, 130.0]; 291.0 [M+4, 31.5]; [M-Me] = 270.3; [M-aryl, Cl fragments] = 174.0. Anal. Calcd. For C_11_H_8_BrClS: C, 45.9; H, 2.80; Found: C, 45.3; H, 2.23.

#### 2,5-Bis(3-chloro-4-fluorophenyl)-3-methylthiophene (**3l**)

Obtained as a yellow solid, mp = 84–86 °C, (100 mg, 56%). ^1^H NMR (CD_3_OD, 500 MHz): δ 7.73 (dd, *J *= 6.6, 2.4 Hz, 2H-aryl), 7.59–7.56 (m, 2H-aryl), 7.27–7.26 (m, 2H-aryl), 7.25 (s, 1H-thiophene), 2.31 (s, 3H-Me); ^13^C NMR (CD_3_OD, 126 MHz): δ 14.5, 117.0 (2C), 118.5, 121.7 (2C), 126.0, 127.5 (m), 128.5, 129.4 (m), 130.0 (m), 133.2, 134.2, 138.3, 158.5 (m), EI/MS *m/z* (%): 356.0 [M+H]; 358.0 [M+2, 65.0]; 360.0 [M+4, 10.6]; 319.0 [M-Me, F fragments], 300.0 [(M+4), Me, 2F fragments]. Anal. Calcd. For C_17_H_10_Cl_2_F_2_S: C, 57.4, H, 2.84; Found: C, 57.0, H, 2.82.

#### 2,5-Bis(4-methoxyphenyl)-3-methylthiophene (**3m**)

Obtained as a brown solid, mp = 90–91 °C, (90 mg, 58%). ^1^H NMR (CD_3_OD, 500 MHz): δ 7.51 (d*, J *= 9.0, 4H-Aryl), 7.38 (d*, J *= 9.0, 4H-Aryl), 7.07 (s, 1H-thiophene), 3.81 (s, 6H-OMe), 2.17 (s, 3H-Me); ^13^C NMR (CD_3_OD, 126 MHz): δ 14.2, 55.2 (2C), 114.0 (4C), 126.0 (2C), 126.4, 128.5 (4C), 133.0, 134.2, 138.0, 160.6 (2C), EI/MS *m/z* (%): 311.0 [M+H]; 295.2 [M-Me]; 203.4 [M-Aryl, OMe fragments]; Anal. Calcd. For C_19_H_18_O_2_S: C, 73.5, H, 5.84; Found: C, 73.0, H, 5.82.

#### 3-Methyl-2,5-bis(4-(methylthio)phenyl)thiophene (**3n**)

Obtained as off-white solid, mp = 160–161 °C, (75 mg, 44%). ^1^H NMR (CD_3_OD, 500 MHz): δ 7.41 (d, *J *= 8.0, 4H-Aryl), 7.31 (d, *J *= 8.5, 4H-Aryl), 7.21 (s, 1H-thiophene), 2.51 (s, 6H-SMe), 2.31 (s, 3H-Me); ^13^C NMR (CD_3_OD, 126 MHz): δ 14.8 (2C), 15.1, 126.5, 127.4 (4C), 127.6 (4C), 130.0 (2C), 133.0, 134.6, 138.0, 139.4 (2C), EI/MS *m/z* (%): 343.9 [M+H]; [M-Me]^+^ = 327.0, [M-Aryl, 2-SMe]^+^ = 173.0. Anal. Calcd. For C_19_H_18_S_3_: C, 66.6; H, 5.30; Found: C, 66.4; H, 5.70%.

#### 2,5-Bis(3,5-dimethylphenyl)-3-methylthiophene (**3o**)

Obtained as colorless oil, (45 mg, 29%). ^1^H NMR (CD_3_OD, 500 MHz): δ 7.21–6.98 (m, 6H-aryl), 6.94 (s, 1H-thiophene), 2.34 (s, 12H-Me), 2.14 (s, 3H-Me); ^13^C NMR (CD_3_OD, 126 MHz): δ 14.2, 21.6 (4C), 126.2, 127.3 (4C), 130.6 (2C), 133.0, 133.8 (2C), 134.0, 138.2, 138.9 (4C), EI/MS *m/z* (%):307.0 [M+H]; [M-Me]^+^= 291.0; [M-2Me]^+^ = 276.0. [M-5Me]^+^ = 231.0. Anal. Calcd. For C_21_H_22_S: C, 82.3, H, 7.24; Found: C, 82.1, H, 7.82.

#### 2,5-Bis(2,3-dichlorophenyl)-3-methylthiophene (**3p**)

Obtained as brown solid, mp = 110–111 °C, (105 mg, 53%). ^1^H NMR (CD_3_OD, 500 MHz): δ 7.52 (dd, *J *= 7.8, 1.2 Hz, 2H-aryl), 7.47–7.46 (m, 2H-aryl), 7.34–7.30 (m, 2H-aryl), 7.10 (s, 1H-thiophene), 2.20 (s, 3H); ^13^C NMR (CD_3_OD, 126 MHz): δ 15.5, 126.4, 127.2 (2C), 127.7 (2C), 130.2 (2C), 131.4 (2C), 133.2, 133.8 (2C), 134.5, 138.3 (3C), EI/MS *m/z* (%):389.0 [M+H^+^]; 391.0 [M+2, 131.0]; 393.0 [M+4), 63.9]; 395.0 [M+6, 14.0]; 397.0 [M+8), 1.2]; [M^+^-2Cl fragments] = 316.0; [M^+^-3Cl fragments] = 281.0; Anal. Calcd. For C_17_H_10_Cl_4_S: C, 51.6, H, 2.60; Found: C, 51.1, H, 2.82.

### Computational methods

By using Gaussian 09 software [40] all simulations were performed and visualization of results was accomplished with Gauss view 05 [41]. All compounds geometries (**3a**–**3p**) were optimized by using B3LYP/6-31G(d,p) basis set at DFT level of theory. Frequency calculations at same level of theory proved true optimization (where no imaginary frequency was observed). Frontier molecular orbital (FMOs) analysis and molecular electrostatic potential (MEP) were carried out at same basis set as used for optimization.

## Pharmacology

### General procedure for antioxidant potential of synthesized compounds by DPPH radical scavenging activity

The DPPH radical scavenging was determined by following the reported method [42]. In the reaction mixture 50 µg/ml of test sample and 1 ml of DPPH (2,2-diphenyl-1-picrylhydrazyl) solution (90 μM) was added and mixture volume was made up to 3 ml. Then incubation of mixture was done at rt for 1 h and absorbance of solution was observed at 515 nm. Sample that contained only methanol was used as blank. Percentage DPPH radical scavenging was calculated by following formula:

$$\% {\text{DPPH}}\,{\text{radical}}\,{\text{scavenging}}\,{\text{activity}} = \left( {\frac{{A_{\text{c}} - A_{\text{s}} }}{{A_{\text{c}} }}} \right) \times 100$$

where, *A*_s_ = absorbance of sample and *A*_c_ = absorbance of control (DPPH solution in methanol without sample).

### General procedure for Antiurease activity

Firstly, phosphate buffer (200 µl, ~ pH = 7) having one unit of enzyme followed by addition of phosphate buffer (230 μl) and stock solution (20 μl) (thiourea or test sample). The mixture was shaked well and at 25 °C it was incubated for 5 min. After this, 400 µl of urea stock (20 mM) solution was added in every sample tube. With no urea solution the calibration mixture was prepared and positive control solution was prepared with no thiourea solution. Then prepared sample solutions were incubated at 40 °C (for 10 min). After this the phenol hypochlorite reagent (1150 μl) was added. For formation of complex and colour development the tubes were further incubated for 25 min at 56 °C. After cooling a blue colour complex appeared and absorbance was observed at 625 nm and % inhibition was calculated by the following formula:

$$\% {\text{age}}\,{\text{inhibition}} = 100 - ({\text{O}} . {\text{D}}\,{\text{of}}\,{\text{test}}\,{\text{sample}}/{\text{O}} . {\text{D}}\,{\text{of}}\,{\text{control}}) \times 100\,$$

The IC_50_ values were determined using the EZ-fit kinetic data base [43, 44].

### General procedure for antibacterial activity

The antibacterial activity of novel molecules was carried out by following already reported method [45] against Gram positive (*Staphylococcus aureus, Bacillus subtilis*) and Gram negative (*Pseudomonas aeruginosa*, *Escherichia coli*, *Salmonella typhi, Shigella dysenteriae*) strains. The bacterial strains were provided by Agha Khan University of Karachi, Pakistan. Streptomycin (50 µg/ml) was used as the positive control. Activity was determined by 96 well plate method. In every well sterilized broth (175 µl) was added and glycerol stock (5.0 µl) bacterial strain was inoculated. The initial absorbance reading maintained between 0.12 and 0.19 and in an incubator bacteria allowed to grow overnight. After 12 h, test sample (20 µl) was added in wells (sample conc was 20 µl/well). The 96 well plates were further incubated (at 37 °C) for 24 h. After incubation the absorbance at 630 nm was observed by using Elisa reader. The difference in absorbance was used as bacterial growth index. Percentage inhibition of bacterial growth was determined by the following formula:

$${\text{Inhibition}}\;(\% ) = \frac{{{\text{O}} . {\text{D}}\,{\text{of}}\,{\text{positive}}\,{\text{control}} - {\text{O}} . {\text{D}}\,{\text{of}}\,{\text{sample}}}}{{{\text{O}} . {\text{D}}\,{\text{of}}\,{\text{positive}}\,{\text{control}}}} \times 100$$

## Conclusion

For the synthesis of some thiophene based pharmaceutically important compounds simple, mild, scalable protocols were developed. The optimized method exhibit enhanced substrate scope and expanded functional group compatibility allowing the synthesis of bundle of novel thiophene based structures in significant yields. Frontier molecular orbitals (FMOs) analysis revealed that **3n** is most reactive having HOMO–LUMO band gap 3.89 eV, whereas HOMO–LUMO band gap for **3p** found 4.67 eV, and is most stable among all. The MEP investigation provided us the idea about the electro and nucleophilic nature of synthesized compounds, and it was envisaged that dispersion of electronic density is highly dependent on nature of groups attached to the aromatic ring. The compounds were screened for biological activities (antibacterial, antiurease and antioxidant). All the tested compounds showed promising biological activities. In light of this research it is concluded that synthesized thiophene derivatives might be a potential source of therapeutic agents. Future investigations in this dimension will provide new visions towards development of novel pharmaceutically important drugs. And these compounds may also be used as intermediates in preparation of fine chemicals for industrial purposes.

## Additional file

**Additional file 1: Figure S1.** HOMO/LUMO surfaces of compounds (**3b**–**3p**). **Table S1.** ESP values of compounds (**3a**–**3p**).
