# Supplementary material for: Palladium(0) catalyzed Suzuki cross-coupling reaction of 2,5-dibromo-3-methylthiophene: selectivity, characterization, DFT studies and their biological evaluations
Source: Chem Cent J. 2018 May 4;12:49. doi: 10.1186/s13065-018-0404-7 (PMC5935605; doi:10.1186/s13065-018-0404-7)
Supplement: Supplementary file 1 — Additional file 1: Figure S1. HOMO/LUMO surfaces of compounds (3b–3p). Table S1. ESP values of compounds (3a–3p). [file 13065_2018_404_MOESM1_ESM.docx]

|  |  |
| --- | --- |
| 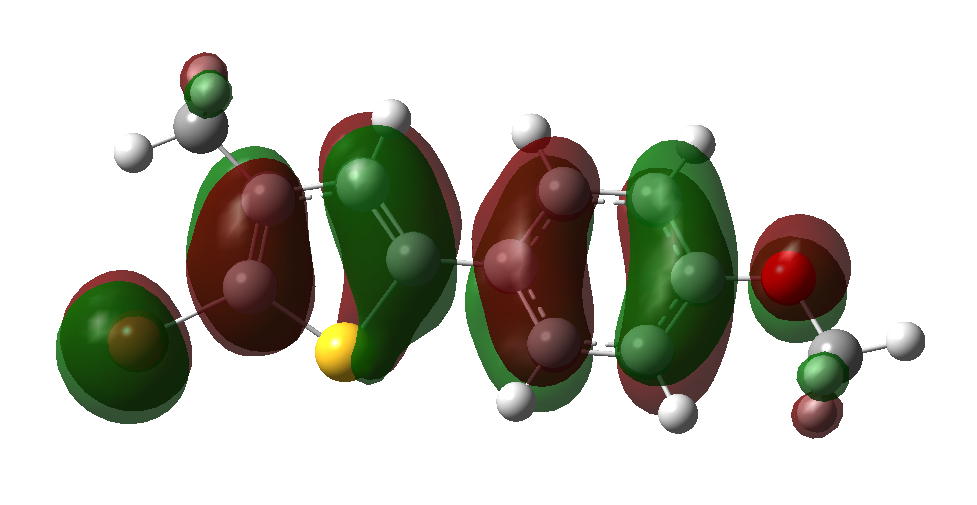  **3b (HOMO)** | 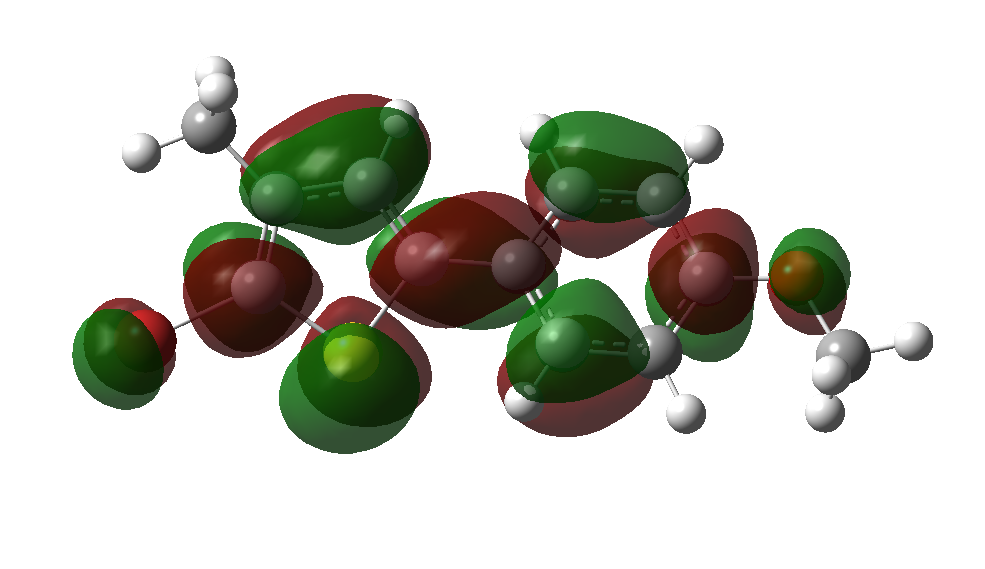**3b (LUMO)** |
| 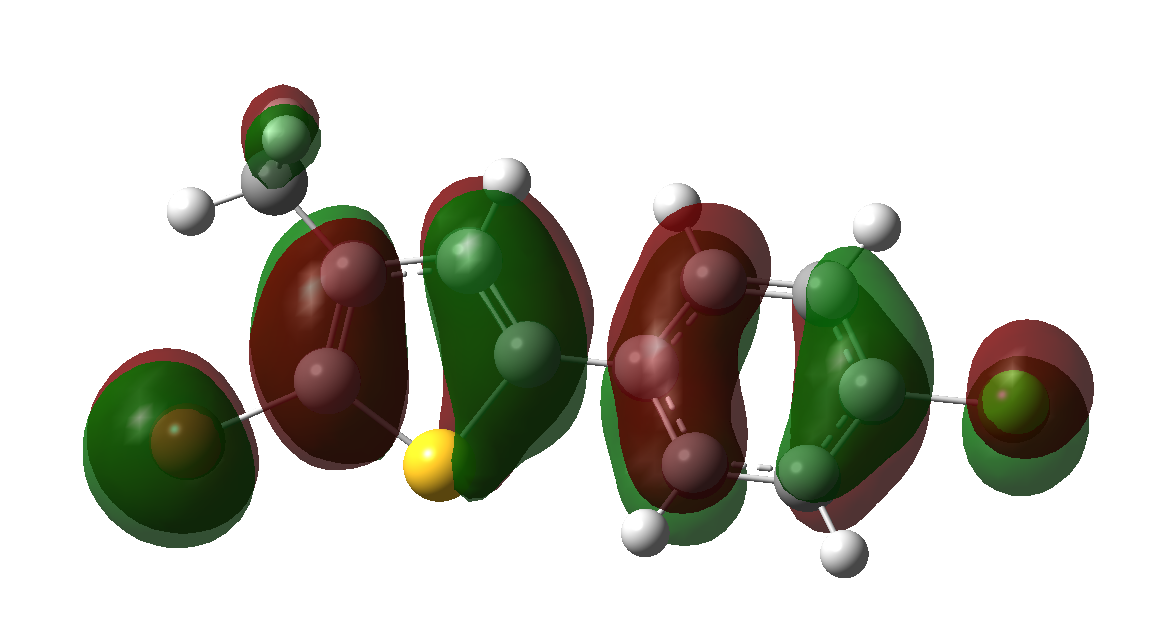  **3c (HOMO)** | 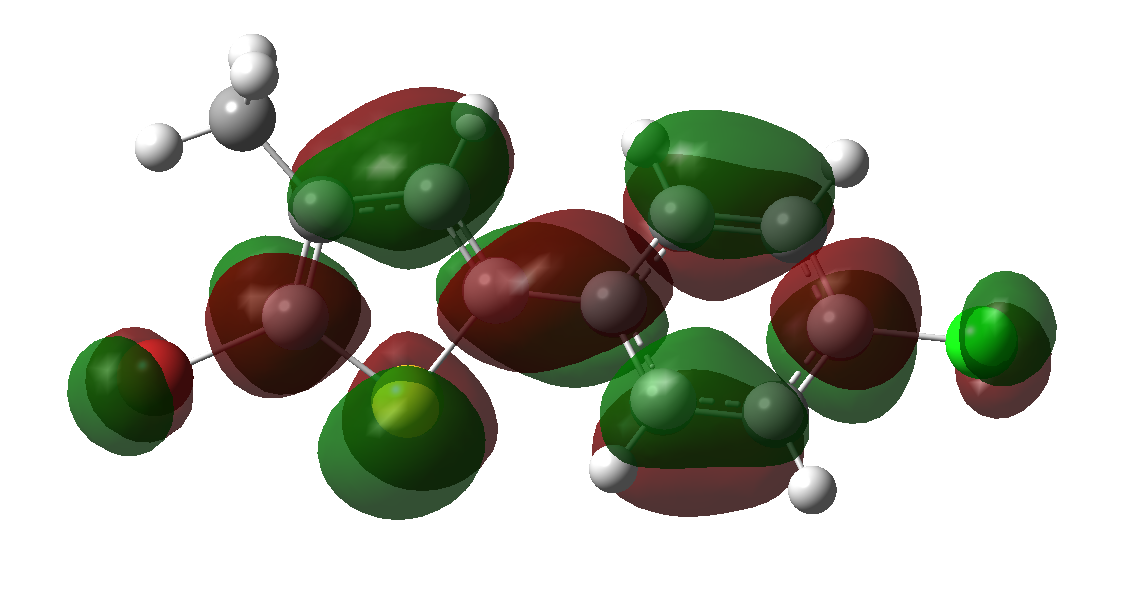  **3c (LUMO)** |
| 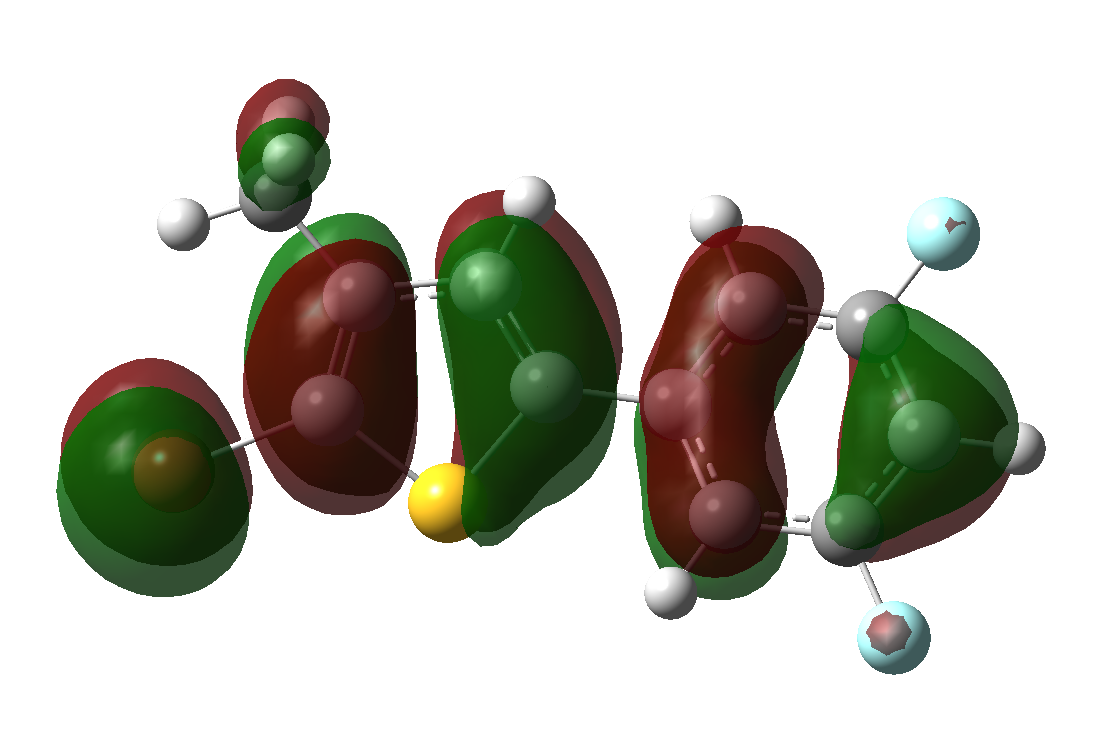  **3d (HOMO)** | 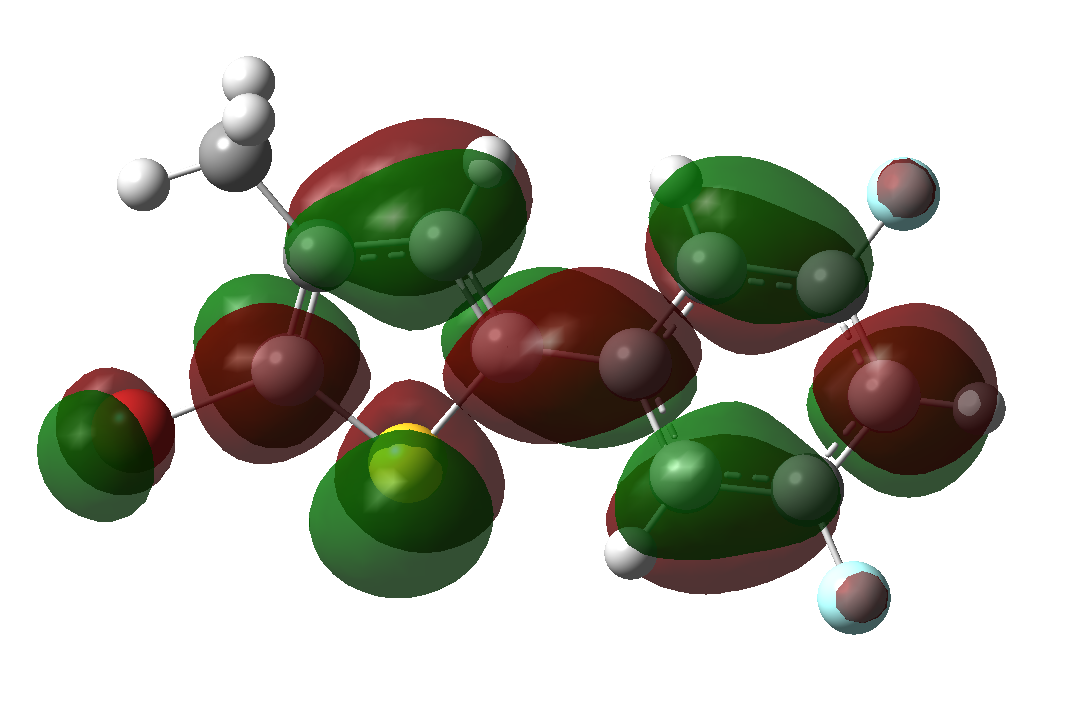  **3d (LUMO)** |
| 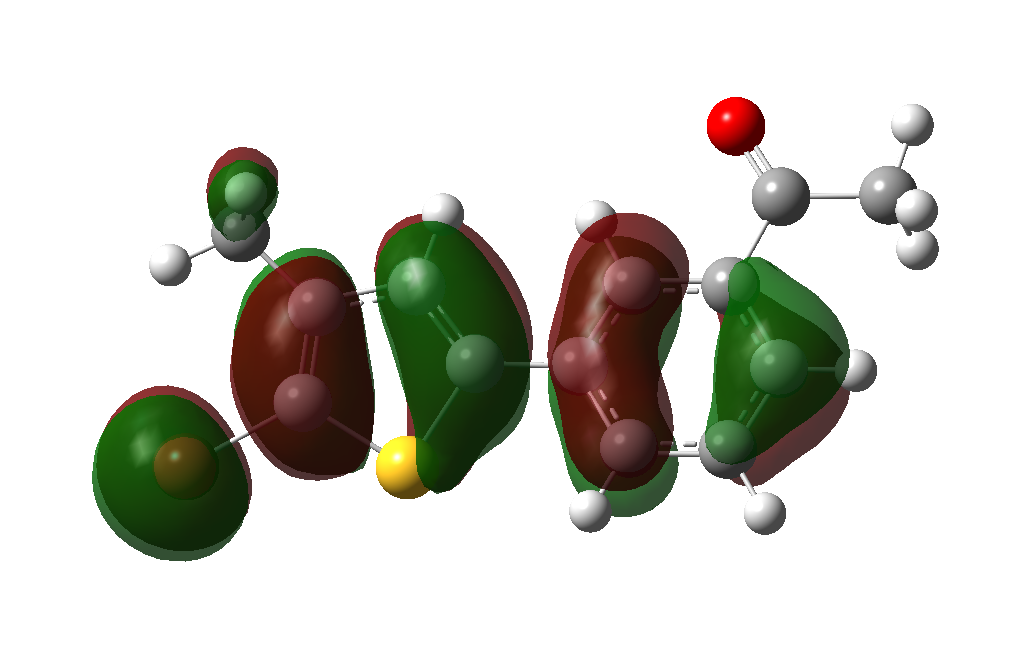  **3e (HOMO)** | 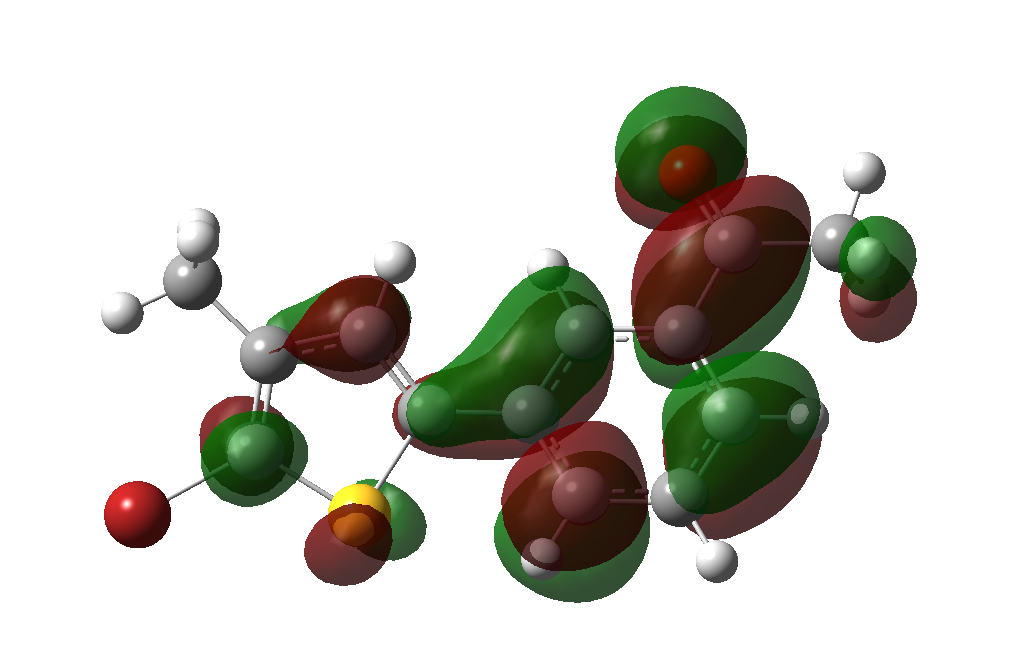  **3e (LUMO)** |
| 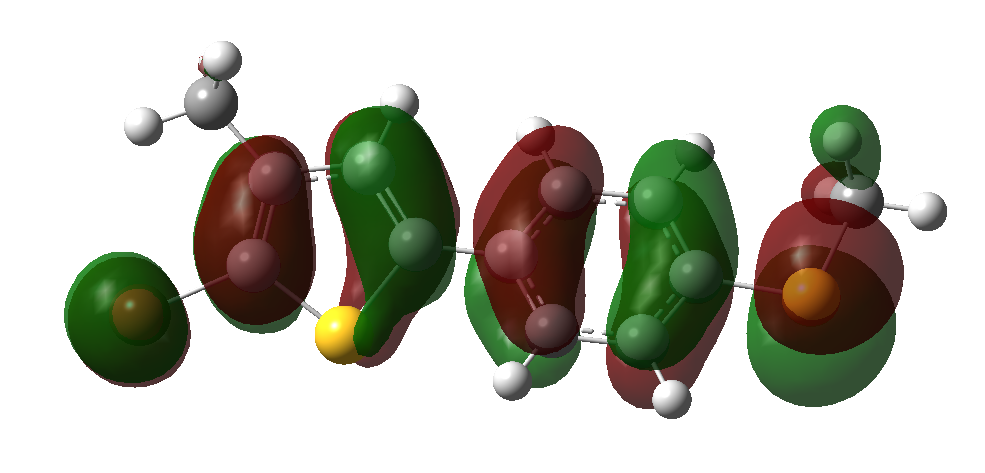  **3f (HOMO)** | 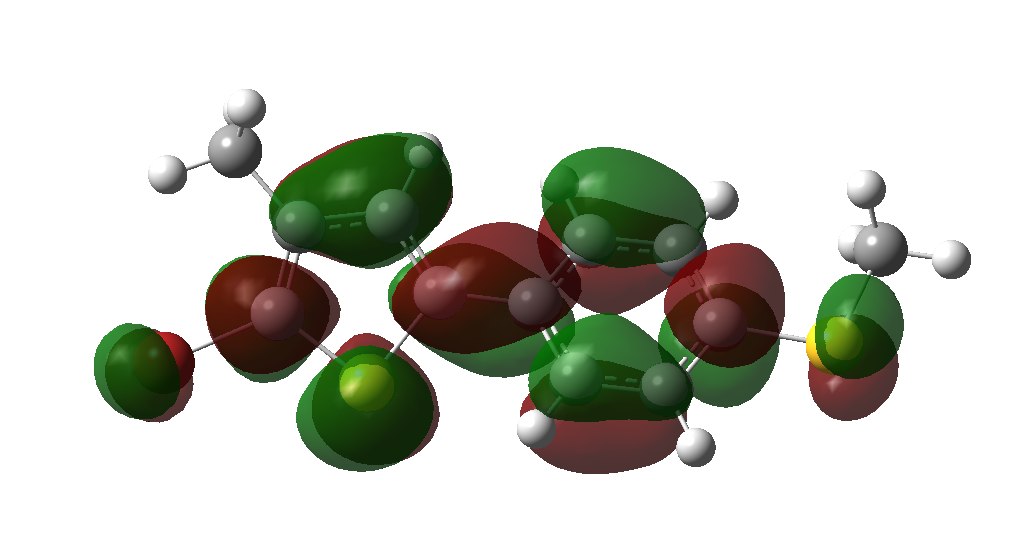  **3f (LUMO)** |
| 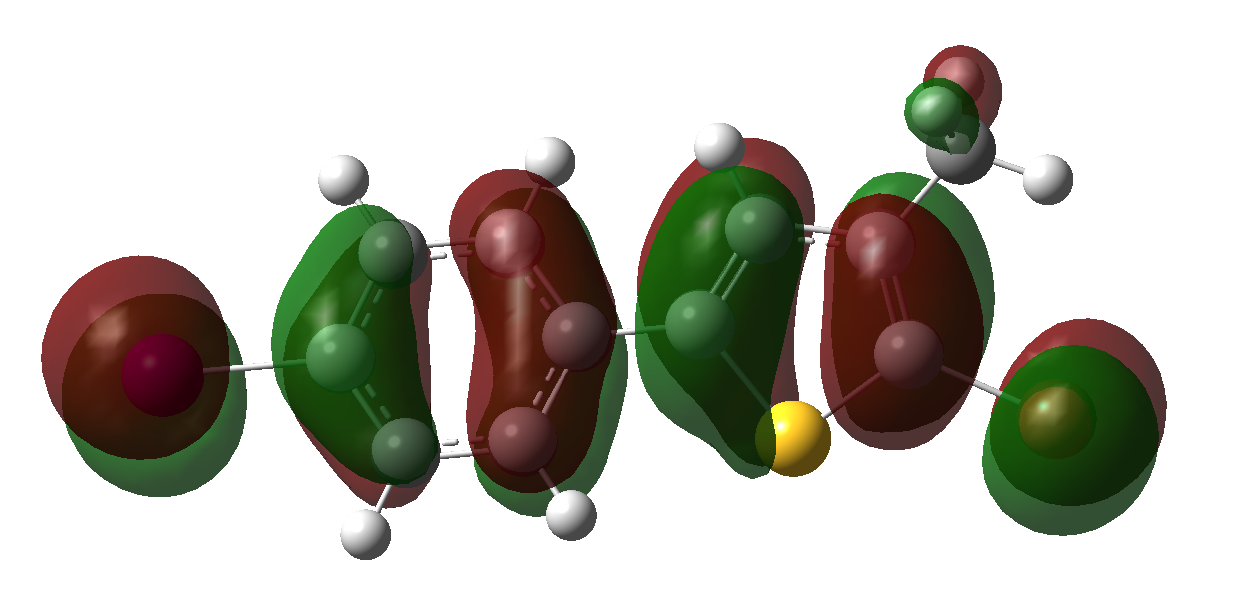  **3g (HOMO)** | 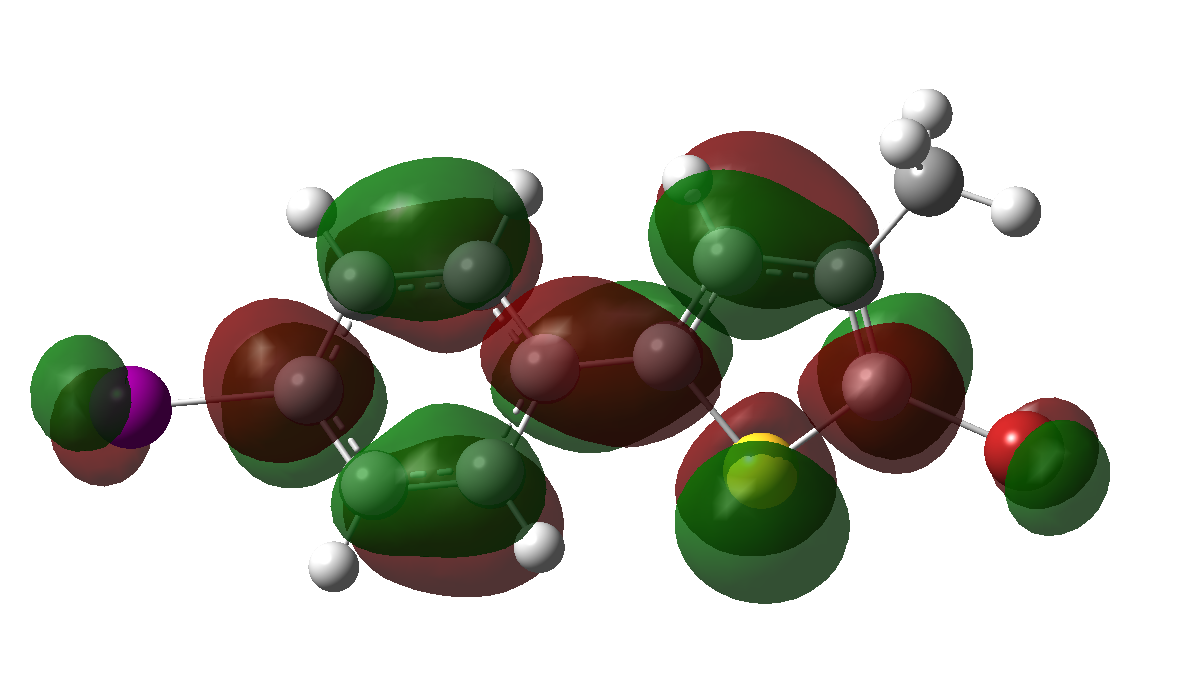  **3g (LUMO)** |
| 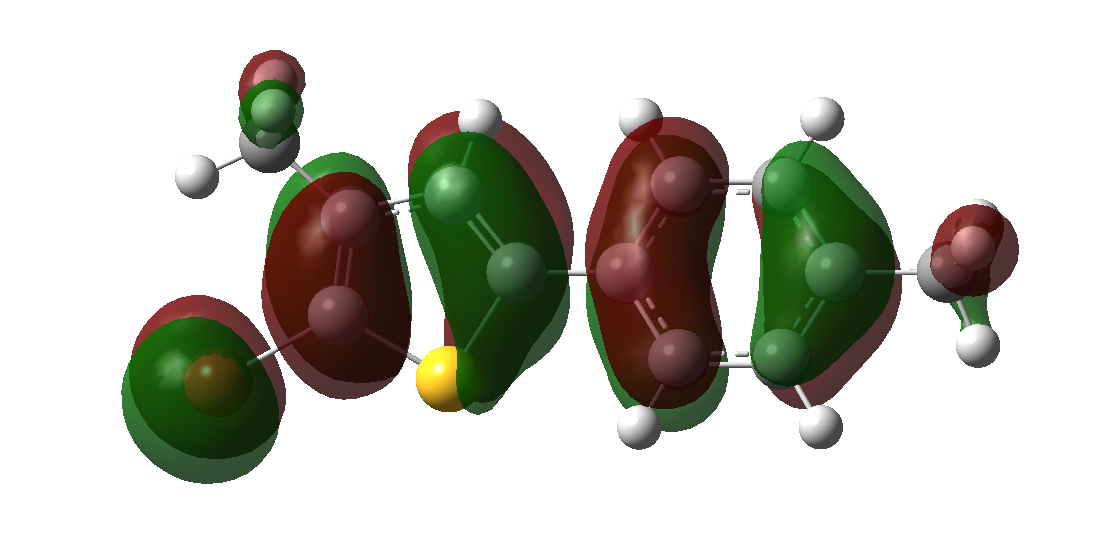  **3h (HOMO)** | 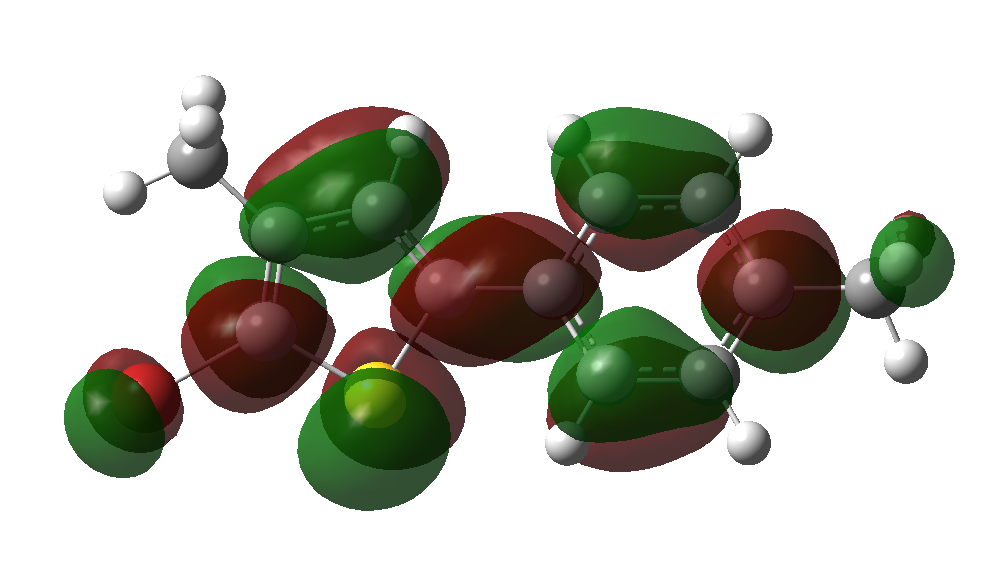  **3h (LUMO)** |
| 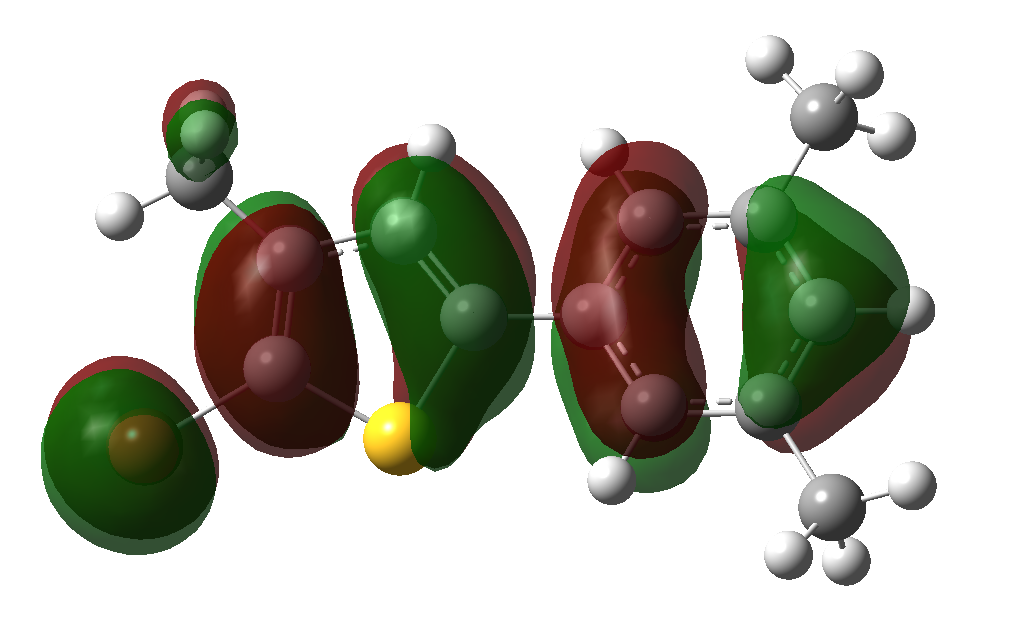  **3i (HOMO)** | 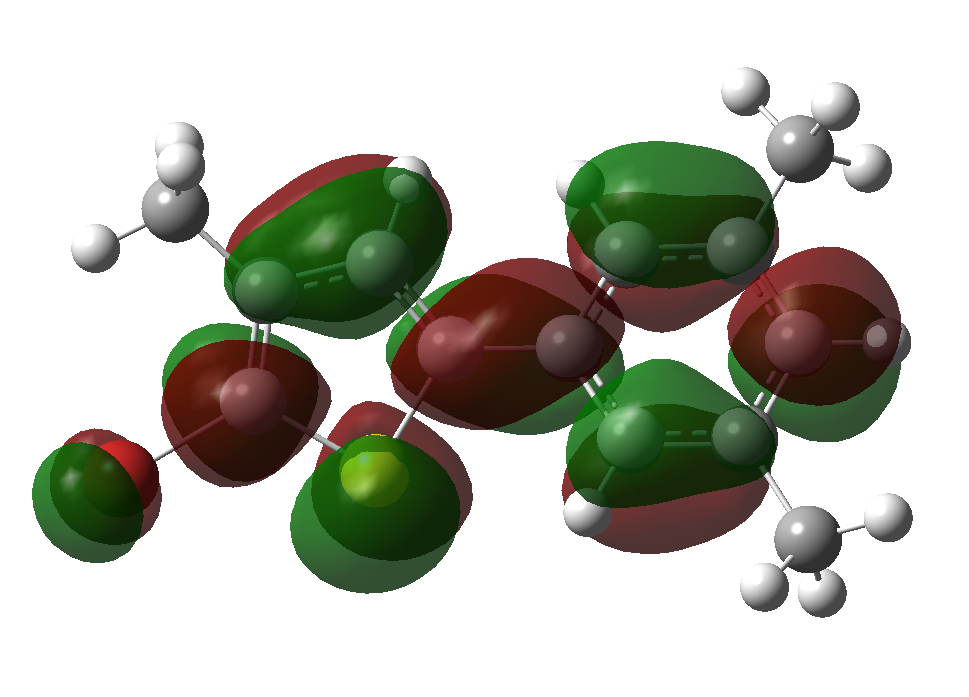  **3i (LUMO)** |
| 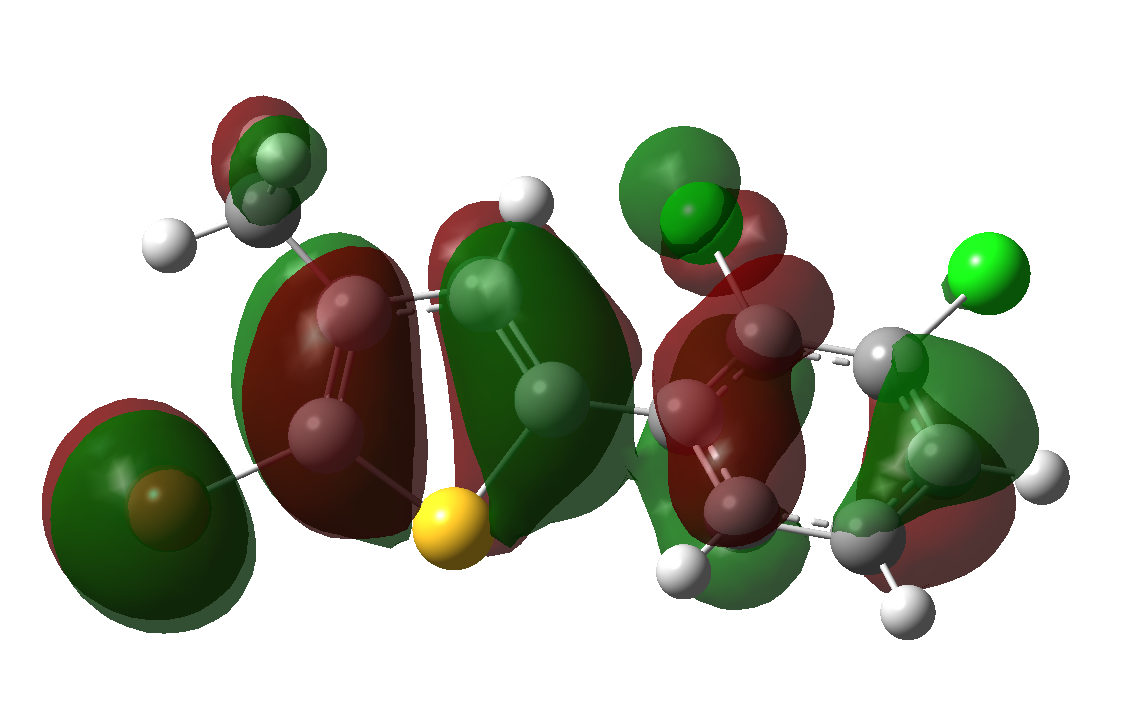  **3j (HOMO)** | 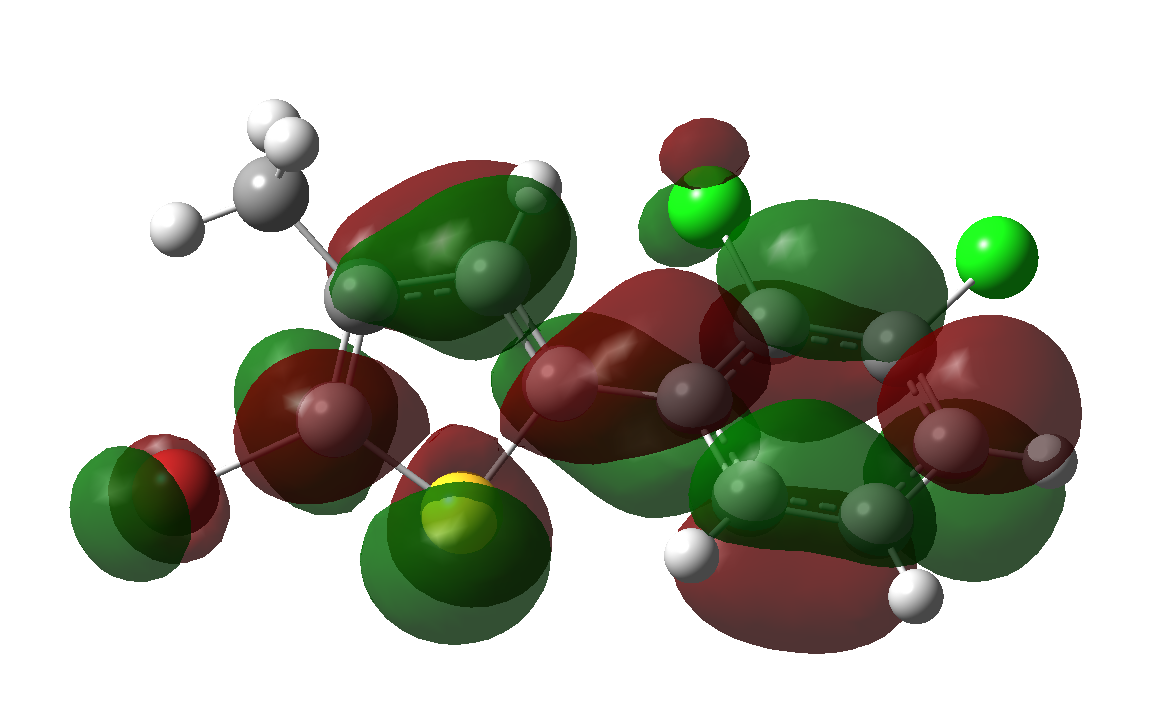  **3j (LUMO)** |
| 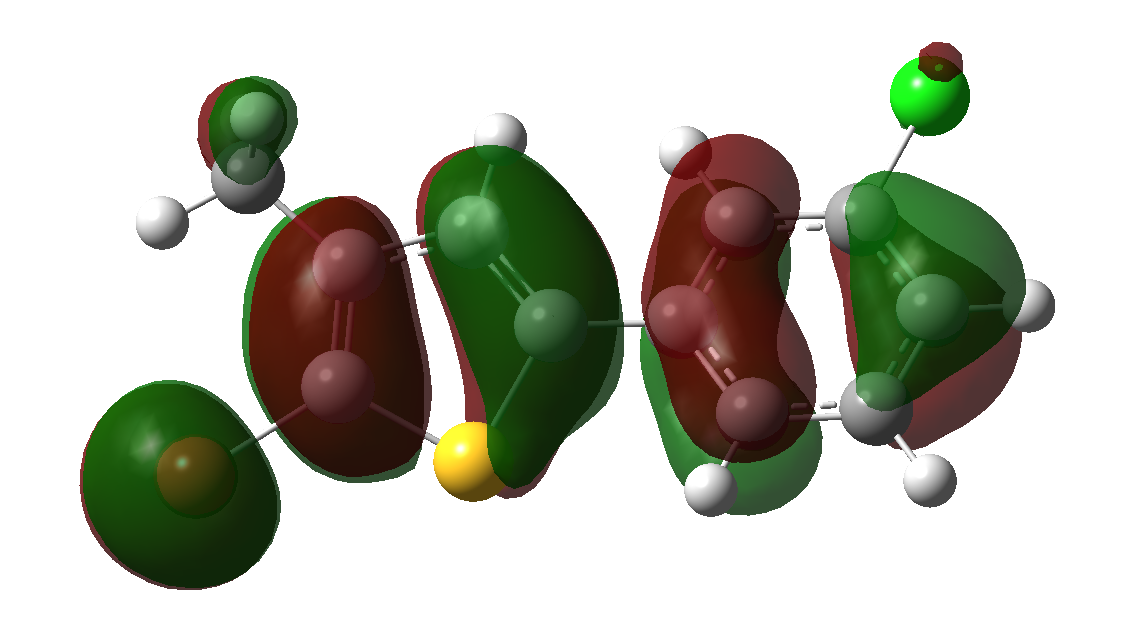  **3k (HOMO)** | 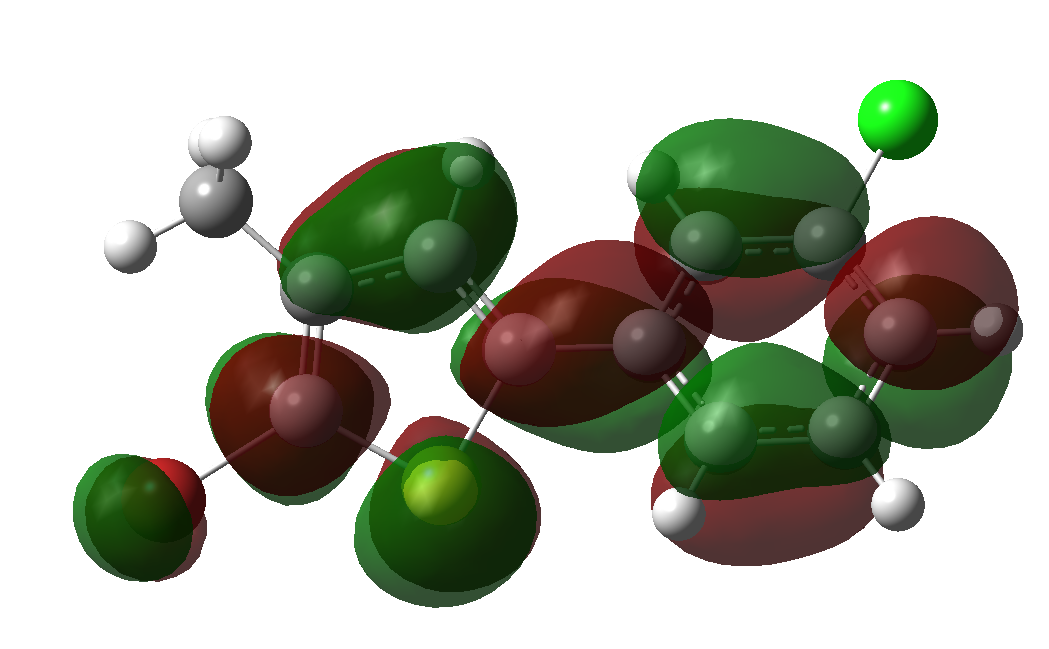  **3k (LUMO)** |
| 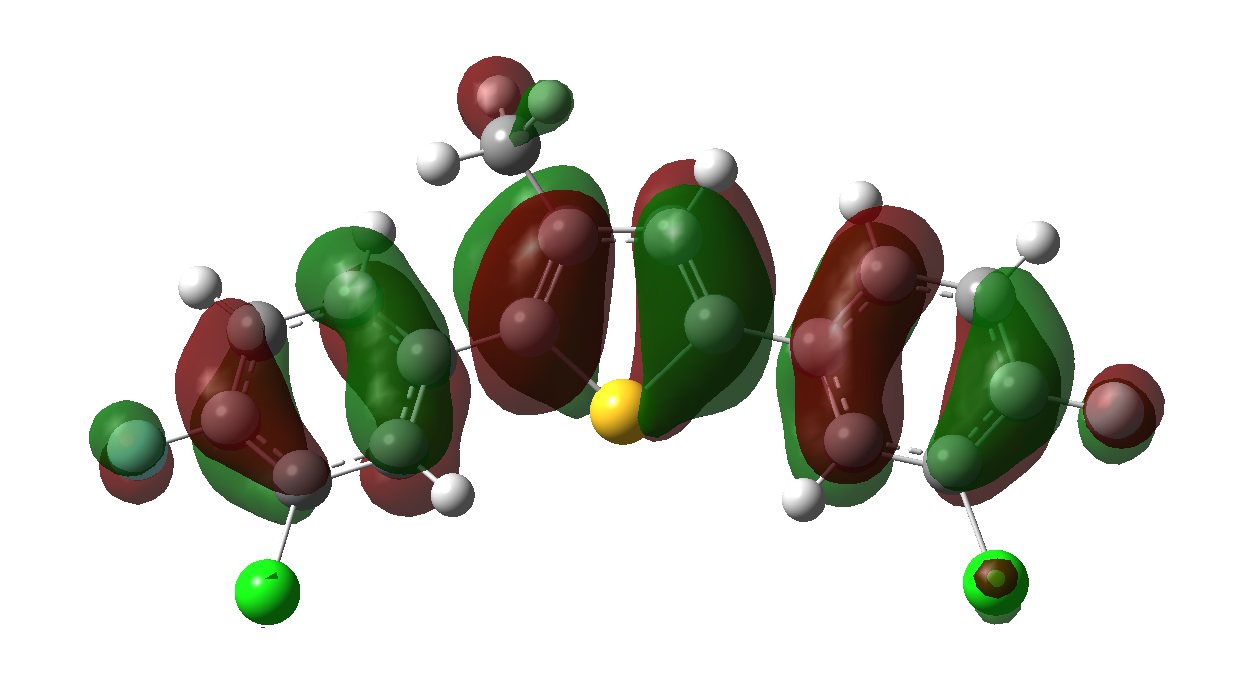  **3l (HOMO)** | 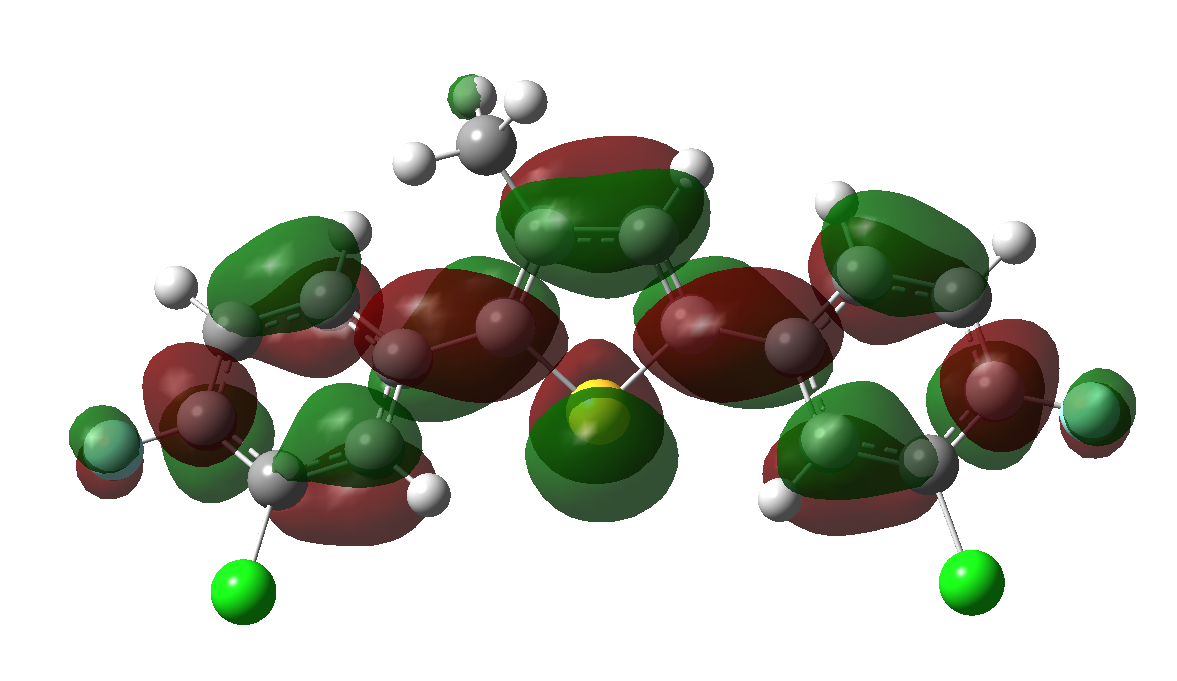  **3l (LUMO)** |
| 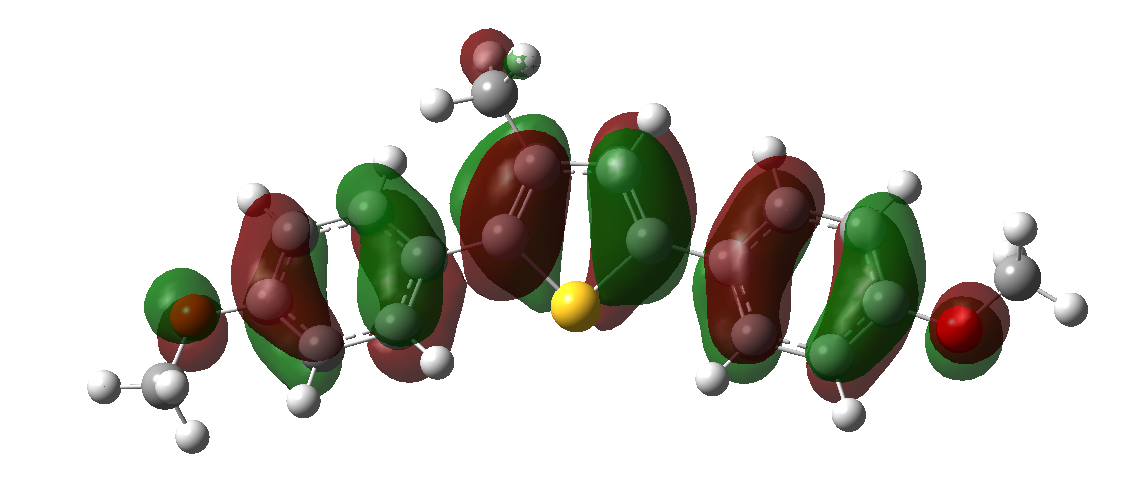  **3m (HOMO)** | 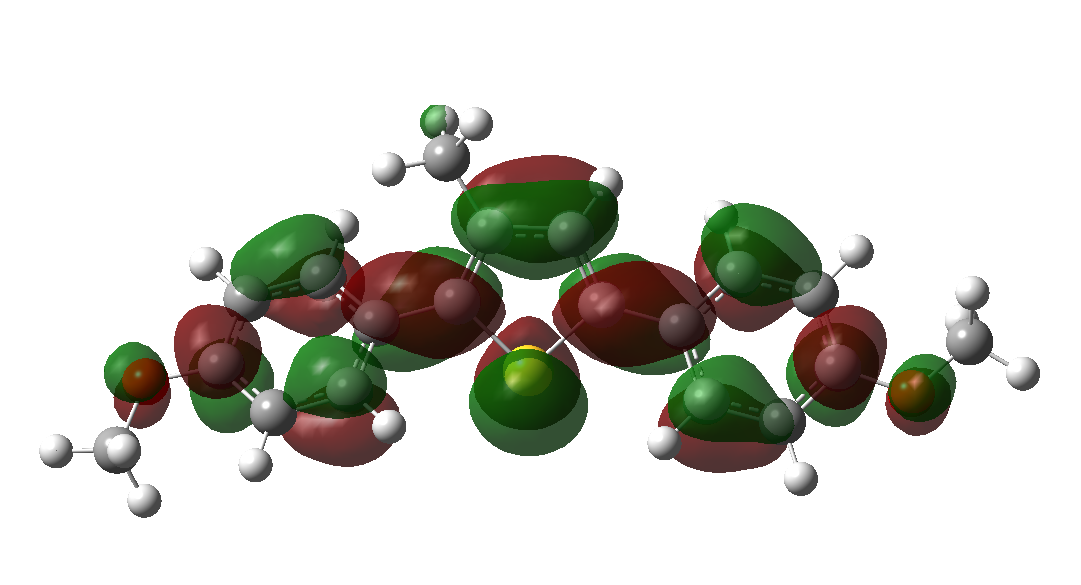  **3m (LUMO)** |
| 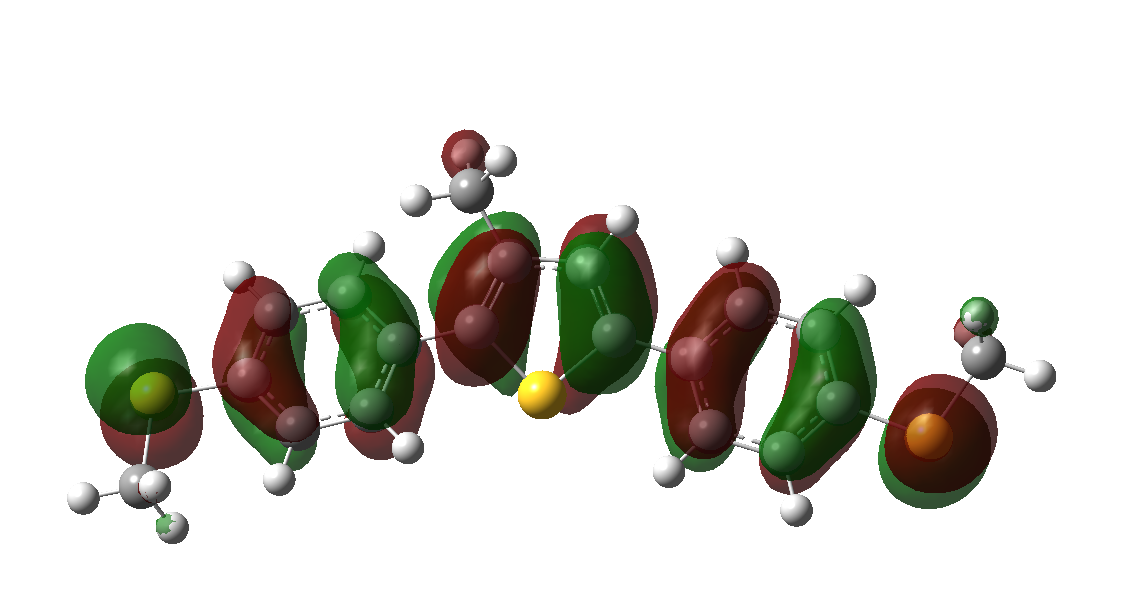  **3n (HOMO)** | 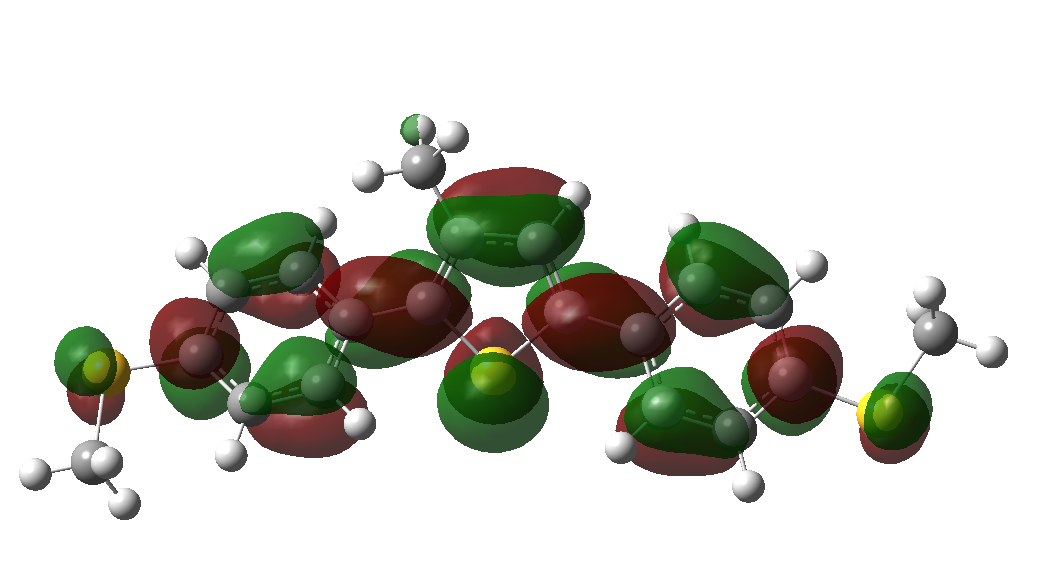  **3n (LUMO)** |
| 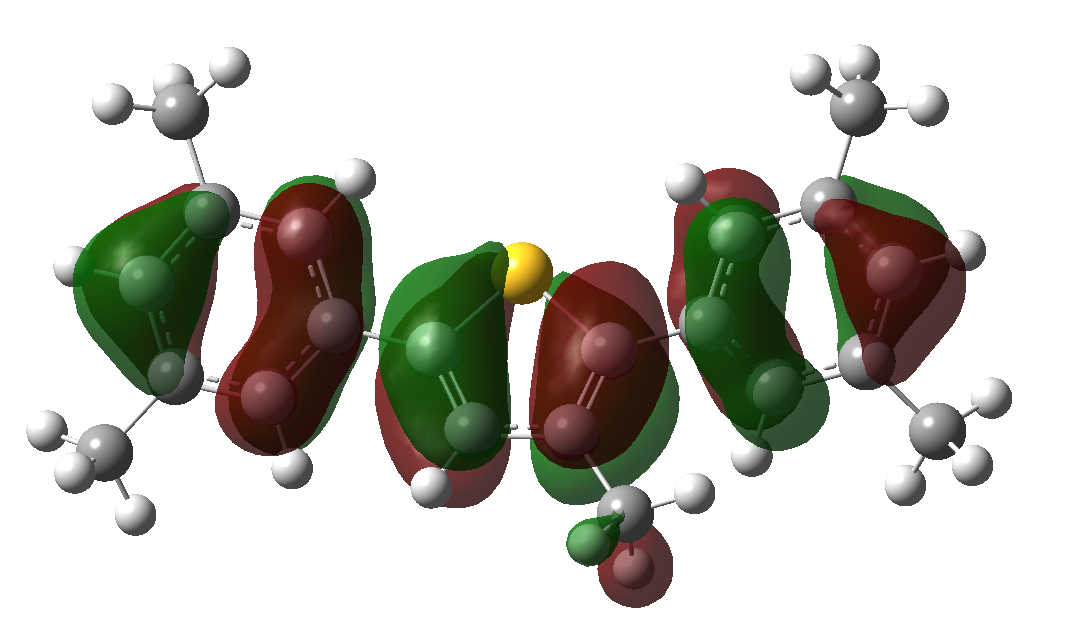  **3o (HOMO)** | 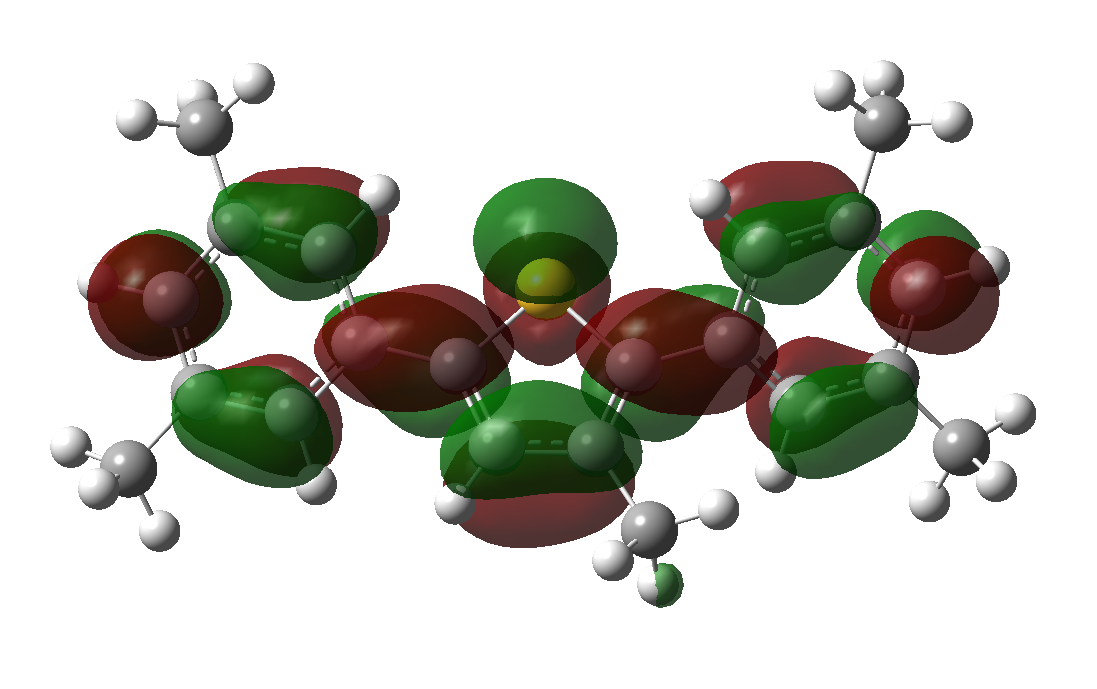  **3o (LUMO)** |
| 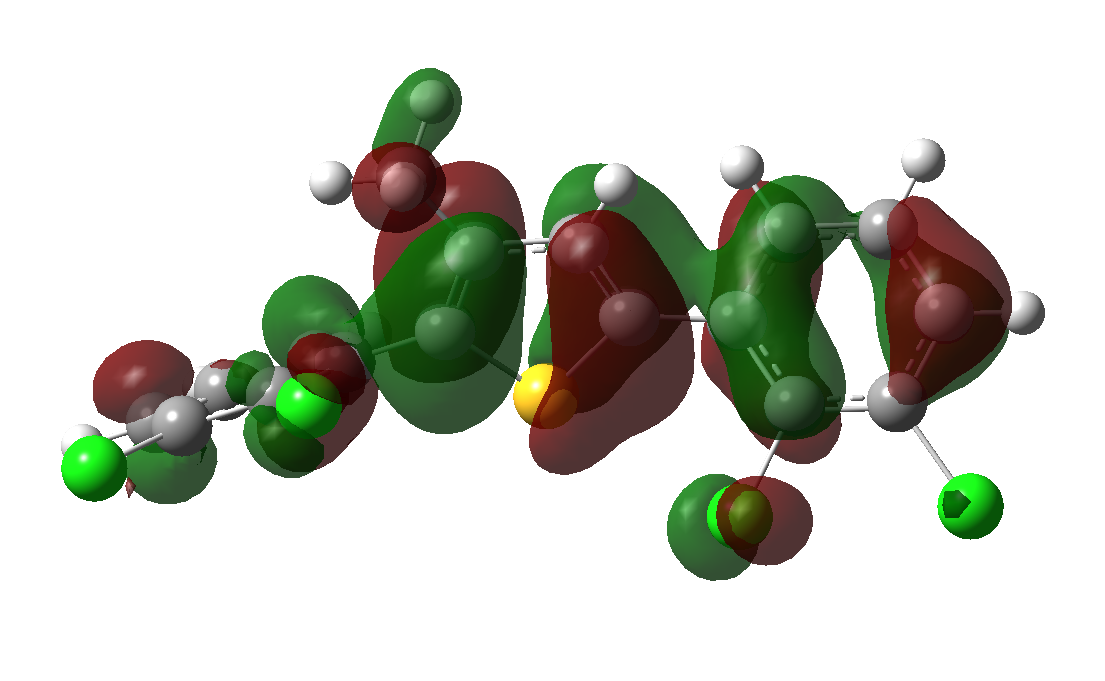  **3p (HOMO)** | 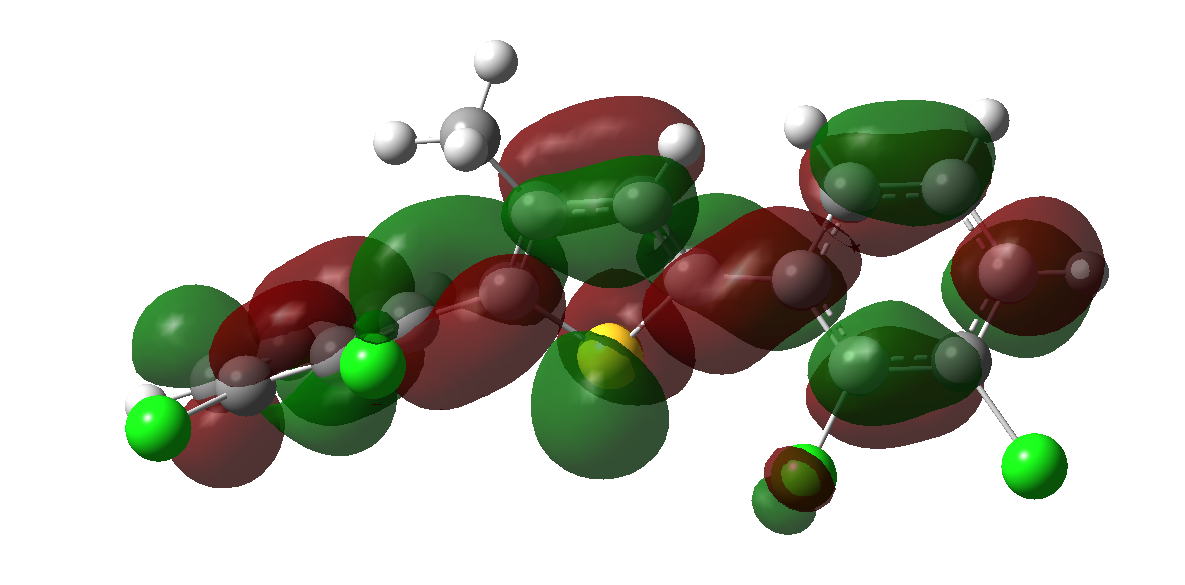  **3p (LUMO)** |

**Figure S1:** HOMO/LUMO surfaces of compounds (**3b**-**3p**)

**Table T1**: ESP values of compounds (**3a**-**3p**)

| Compounds No | **-ve ESP potential (a. u.)** | **+ve ESP potential (a. u.)** |
| --- | --- | --- |
| 3a | -0.0301 | 0.0301 |
| 3b | -0.0264 | 0.0264 |
| 3c | -0.0274 | 0.0274 |
| 3d | -0.0277 | 0.0277 |
| 3e | -0.0481 | 0.0481 |
| 3f | -0.0257 | 0.0257 |
| 3g | -0.0318 | 0.0318 |
| 3h | -0.0204 | 0.0204 |
| 3i | -0.0212 | 0.0212 |
| 3j | -0.0259 | 0.0259 |
| 3k | -0.0244 | 0.0244 |
| 3l | -0.0296 | 0.0296 |
| 3m | -0.0302 | 0.0302 |
| 3n | -0.0256 | 0.0256 |
| 3o | -0.0264 | 0.0264 |
| 3p | -0.0261 | 0.0261 |
|  |  |  |
